# Anisotropic Hardy Spaces of Musielak-Orlicz Type with Applications to Boundedness of Sublinear Operators

**DOI:** 10.1155/2014/306214

**Published:** 2014-03-16

**Authors:** Baode Li, Dachun Yang, Wen Yuan

**Affiliations:** ^1^School of Mathematics and System Sciences, Xinjiang University, Urumqi 830046, China; ^2^School of Mathematical Sciences, Beijing Normal University, Laboratory of Mathematics and Complex Systems, Ministry of Education, Beijing 100875, China

## Abstract

Let *φ* : ℝ^*n*^ × [0, ∞)→[0, ∞) be a Musielak-Orlicz function and *A* an expansive dilation. In this paper, the authors introduce the anisotropic Hardy space of Musielak-Orlicz type, *H*_*A*_^*φ*^(ℝ^*n*^), via the grand maximal function. The authors then obtain some real-variable characterizations of *H*_*A*_^*φ*^(ℝ^*n*^) in terms of the radial, the nontangential, and the tangential maximal functions, which generalize the known results on the anisotropic Hardy space *H*_*A*_^*p*^(ℝ^*n*^) with *p* ∈ (0,1] and are new even for its weighted variant. Finally, the authors characterize these spaces by anisotropic atomic decompositions. The authors also obtain the finite atomic decomposition characterization of *H*_*A*_^*φ*^(ℝ^*n*^), and, as an application, the authors prove that, for a given admissible triplet (*φ*, *q*, *s*), if *T* is a sublinear operator and maps all (*φ*, *q*, *s*)-atoms with *q* < ∞ (or all continuous (*φ*, *q*, *s*)-atoms with *q* = ∞) into uniformly bounded elements of some quasi-Banach spaces *ℬ*, then *T* uniquely extends to a bounded sublinear operator from *H*_*A*_^*φ*^(ℝ^*n*^) to *ℬ*. These results are new even for anisotropic Orlicz-Hardy spaces on ℝ^*n*^.

## 1. Introduction

The theory of Hardy spaces on the Euclidean space ℝ^*n*^ plays an important role in various fields of analysis and partial differential equations (see, e.g., [1–5]). One of the most important applications of Hardy spaces is that they are good substitutes of Lebesgue spaces when *p* ∈ (0, 1]. For example, when *p* ∈ (0, 1], it is well known that Riesz transforms are not bounded on *L* ^*p*^(ℝ^*n*^); however, they are bounded on Hardy spaces *H* ^*p*^(ℝ^*n*^). Moreover, there were several efforts to extend classical Hardy spaces, some of which are weighted anisotropic Hardy spaces [6] associated with general expansive dilations and *A*_*∞*_ Muckenhoupt weights. These Hardy spaces include classical isotropic Hardy spaces of Fefferman and Stein [1], parabolic Hardy spaces of Calderón and Torchinsky [7], and weighted Hardy spaces of García-Cuerva [8] as well as Strömberg and Torchinsky [5] as special cases. Apart from their theoretical consideration, such anisotropic function spaces also play an important role in allowing even more general discrete dilation structures which have originated from the theory of wavelets; see, for example, [9, 10].

On the other hand, as a generalization of *L* ^*p*^(ℝ^*n*^), the Orlicz space was introduced by Birnbaum and Orlicz in [11] and Orlicz in [12]. Since then, the theory of the Orlicz spaces themselves has been well developed and these spaces have been widely used in many branches of analysis (see, e.g., [13–15]). Moreover, as a development of the theory of Orlicz spaces, Orlicz-Hardy spaces and their dual spaces were studied by Strömberg [16] and Janson [17] on ℝ^*n*^ and, quite recently, Orlicz-Hardy spaces associated with divergence form elliptic operators by Jiang and Yang [18].

Let *𝒜*_*q*_(ℝ^*n*^) with *q* ∈ [1, *∞*] denote the class of* Muckenhoupt weights* (see, e.g., [19] for their definitions and properties) and let *φ* be a* growth function* (see [20]) which means that *φ* : ℝ^*n*^ × [0, *∞*)→[0, *∞*) is a Musielak-Orlicz function such that *φ*(*x*, ·) is an Orlicz function and *φ*(·, *t*) is a Muckenhoupt *𝒜*_*∞*_(ℝ^*n*^) weight. It is known that Musielak-Orlicz functions are the natural generalization of Orlicz functions that may vary in the spatial variables (see, e.g., [20–23]). Recently, Ky [20] introduced a new* Musielak-Orlicz Hardy* space *H* ^*φ*^(ℝ^*n*^), via the grand maximal function, and established its atomic characterization. It is known that *H* ^*φ*^(ℝ^*n*^) generalizes both the Orlicz-Hardy space of Strömberg [16] and Janson [17] and the weighted Hardy space *H*_*w*_^*p*^(ℝ^*n*^) with *w* ∈ *𝒜*_*∞*_(ℝ^*n*^) studied by García-Cuerva [8] and Strömberg and Torchinsky [5]. Recall that the motivation to study function spaces of Musielak-Orlicz type comes from their applications to many branches of mathematics and physics (see, e.g., [20, 23–27]). In [20], Ky further introduced the BMO-type space BMO_*φ*_(ℝ^*n*^), which was proven to be the dual space of *H* ^*φ*^(ℝ^*n*^); as an interesting application, Ky proved that the class of pointwise multipliers for BMO(ℝ^*n*^), characterized by Nakai and Yabuta [28, 29], is the dual space of *L* ^1^(ℝ^*n*^) + *H* ^log⁡^(ℝ^*n*^), where *H* ^log⁡^(ℝ^*n*^) denotes the Musielak-Orlicz Hardy space related to the growth function

(1) $$\begin{array}{c} \varphi \left(x,t\right)≔\frac{t}{\log\left(e+\left|x\right|\right)+\log\left(e+t\right)} \end{array}$$

for all *x* ∈ ℝ^*n*^ and *t* ∈ [0, *∞*). It is worth noticing that some special Musielak-Orlicz Hardy spaces appear naturally in the study of the products of functions in BMO(ℝ^*n*^) and *H* ^1^(ℝ^*n*^) (see [25, 26, 30]), the endpoint estimates for the div-curl lemma, and the commutators of singular integral operators (see [25, 30–32]).

Moreover, observe that a distribution in Hardy spaces can be represented as a (finite or infinite) linear combination of atoms (see [33, 34]). Then, the boundedness of linear operators in Hardy spaces can be deduced from their behavior on atoms in principle. However, Meyer et al. [35, page 513] gave an example of *f* ∈ *H* ^1^(ℝ^*n*^) whose norm can not be achieved by its finite atomic decompositions via (1, *∞*, 0)-atoms. Applying this, Bownik [36] showed that there exists a linear functional defined on a dense subspace of *H* ^1^(ℝ^*n*^), which maps all (1, *∞*, 0)-atoms into bounded scalars, but yet can not extend to a bounded linear functional on the whole *H* ^1^(ℝ^*n*^). Let *p* ∈ (0,1] and let *s* be a nonnegative integer not less than *n*(1/*p* − 1). This implies that the uniform boundedness in some quasi-Banach space *ℬ* of a linear operator *T* on all (*p*, *∞*, *s*)-atoms does not generally guarantee the boundedness of *T* from *H* ^*p*^(ℝ^*n*^) to *ℬ*. This phenomenon has also essentially already been observed by Meyer et al. in [37, page 19]. Motivated by [36], via using the Lusin function characterization of Hardy spaces *H* ^*p*^(ℝ^*n*^), Yang and Zhou [38] proved that a *ℬ*_*γ*_-sublinear operator *T* uniquely extends to a bounded *ℬ*_*γ*_-sublinear operator from *H* ^*p*^(ℝ^*n*^) with *p* ∈ (0, 1] to some quasi-Banach space *ℬ* if and only if *T* maps all (*p*, 2, *s*)-atoms into uniformly bounded elements of *ℬ*. Independently, Meda et al. [39] established another more general bounded criterion via using the grand maximal function characterization of *H* ^*p*^(ℝ^*n*^); precisely, they proved that if *T* is a linear operator and maps all (*p*, *q*, *s*)-atoms with *q* < *∞* or all continuous (*p*, *∞*, *s*)-atoms into uniformly bounded elements of a Banach space *ℬ*, then *T* uniquely extends to a bounded linear operator from *H* ^*p*^(ℝ^*n*^) to *ℬ*. This result was further generalized to the weighted anisotropic Hardy spaces in [6], weighted anisotropic product Hardy spaces in [40], and, especially, Hardy spaces of Musielak-Orlicz type by Ky in [20].

There are three goals in this paper. First, we introduce anisotropic Hardy spaces of Musielak-Orlicz type, *H*_*A*_^*φ*^(ℝ^*n*^), via grand maximal functions and characterize these spaces via anisotropic atomic decompositions. These Hardy spaces include classical Hardy spaces *H* ^*p*^(ℝ^*n*^) of Fefferman and Stein [1], weighted anisotropic Hardy spaces of Bownik [6], and Hardy spaces of Musielak-Orlicz type of Ky [20].

The second goal is to obtain some new real-variable characterizations of *H*_*A*_^*φ*^(ℝ^*n*^) in terms of the radial, the nontangential, and the tangential maximal functions via some bounded estimates of the truncated maximal function pointwise or in anisotropic Musielak-Orlicz spaces which are motivated by [9, Section 7]. These real-variable characterizations of *H*_*A*_^*φ*^(ℝ^*n*^) coincide with the known best results, when *H*_*A*_^*φ*^(ℝ^*n*^) is the anisotropic Hardy space *H*_*A*_^*p*^(ℝ^*n*^), with *p* ∈ (0,1] (see [9, Theorem 7.1]), or new even in its weighted variant.

The third goal is to generalize the result of Meda et al. [39] to the present setting. More precisely, we prove the existence of finite atomic decompositions achieving the norm in dense subspaces of *H*_*A*_^*φ*^(ℝ^*n*^). As an application, we prove that, for a given admissible triplet (*φ*, *q*, *s*) (see Definition 30 below), if *T* is a *ℬ*_*γ*_-sublinear operator and maps all (*φ*, *q*, *s*)-atoms with *q* < *∞* (or all continuous (*φ*, *q*, *s*)-atoms with *q* = *∞*) into uniformly bounded elements of some quasi-Banach spaces *ℬ*, then *T* uniquely extends to a bounded *ℬ*_*γ*_-sublinear operator from *H*_*A*_^*φ*^(ℝ^*n*^) to *ℬ*. These results are new even for the anisotropic Hardy-Orlicz spaces on ℝ^*n*^.

This paper is organized as follows. In Section 2, we first recall some notation and definitions concerning Musielak-Orlicz functions, expansive dilations, and Muckenhoupt weights. Then we introduce the anisotropic Hardy spaces of Musielak-Orlicz type, *H*_*A*_^*φ*^(ℝ^*n*^), via grand maximal functions, and some basic properties of these spaces are also presented. In Section 3, we obtain some new real-variable characterizations of *H*_*A*_^*φ*^(ℝ^*n*^) via the radial, the nontangential, and the tangential maximal functions.  Section 4 is devoted to generalizing the Calderón-Zygmund decomposition associated to weighted anisotropic Hardy spaces in [6] to the more general spaces *H*_*A*_^*φ*^(ℝ^*n*^). Applying this, in Section 5, we introduce the anisotropic atomic Hardy spaces of Musielak-Orlicz type, *H*_*A*_^*φ*,*q*,*s*^(ℝ^*n*^), for any admissible triplet (*φ*, *q*, *s*), and further prove that, for any admissible triplet (*φ*, *q*, *s*),

(2) $$\begin{array}{c} H_{A}^{\varphi }\left({\mathbb{R}}^{n}\right)=H_{A}^{\varphi ,q,s}\left({\mathbb{R}}^{n}\right) \end{array}$$

with equivalent norms (see Theorem 40 below). Moreover, in Section 6.1, we prove that ||·||_*H*_*A*,fin_^*φ*,*q*,*s*^(ℝ^*n*^)_ and ||·||_*H*_*A*_^*φ*^(ℝ^*n*^)_ are equivalent quasinorms on *H*_*A*,fin_^*φ*,*q*,*s*^(ℝ^*n*^) when *q* < *∞* and on *H*_*A*,fin_^*φ*,*q*,*s*^(ℝ^*n*^)∩*ℂ*(ℝ^*n*^) when *q* = *∞*, where *H*_*A*,fin_^*φ*,*q*,*s*^(ℝ^*n*^) denotes the space of all finite linear combinations of multiples of (*φ*, *q*, *s*)-atoms. In Section 6.2, we obtain criteria for boundedness of sublinear operators in *H*_*A*_^*φ*^(ℝ^*n*^) (see Theorem 44 below). The results in Section 6 are also new even for the anisotropic Hardy-Orlicz spaces on ℝ^*n*^.

Finally, we make some conventions on notation. Let *ℕ*≔{1, 2,…} and let *ℤ*_+_≔{0} ∪ *ℕ*. Denote by *𝒮*(ℝ^*n*^) the* space of all Schwartz functions* and *𝒮*′(ℝ^*n*^) the* space of all tempered distributions*. For any *α*≔(*α*_1_,…, *α*_*n*_) ∈ *ℤ*_+_^*n*^, |*α* | ≔*α*_1_ + ⋯+*α*_*n*_ and ∂^*α*^≔(∂/∂*x*_1_)^*α*_1_^ ⋯ (∂/∂*x*_*n*_)^*α*_*n*_^. Throughout the whole paper, we denote by *C* a* positive constant* which is independent of the main parameters, but it may vary from line to line. The* symbol* *D*≲*F* means that *D* ≤ *CF*. If *D*≲*F* and *F*≲*D*, we then write *D* ~ *F*. If *E* is a subset of ℝ^*n*^, we denote by *χ*_*E*_ its* characteristic function*. For any *a* ∈ ℝ, ⌊*a*⌋ denotes the* maximal integer* not larger than *a*.

## 2. Anisotropic Hardy Spaces of Musielak-Orlicz Type

In this section, we introduce anisotropic Hardy spaces of Musielak-Orlicz type via grand maximal functions and give out some basic properties.

First let us recall some notation for Orlicz functions; see, for example, [20]. A function *ϕ* : [0, *∞*)→[0, *∞*) is called an* Orlicz function* if it is nondecreasing and *ϕ*(0) = 0, *ϕ*(*t*) > 0 if *t* > 0, and lim⁡_*t*→*∞*_ *ϕ*(*t*) = *∞*. Observe that, differently from the classical Orlicz functions being convex, the Orlicz functions in this paper may not be convex. An Orlicz function *ϕ* is said to be of* lower *(resp.*, upper*)* type p* with *p* ∈ (−*∞*, *∞*), if there exists a positive constant *C* such that, for all *t* ∈ [0, *∞*) and *s* ∈ (0,1) (resp., *s* ∈ [1, *∞*)),

(3) $$\begin{array}{c} \phi \left(st\right)\leq Cs^{p}\phi \left(t\right). \end{array}$$

Given the function *φ* : ℝ^*n*^ × [0, *∞*)→[0, *∞*) such that, for any *x* ∈ ℝ^*n*^, *φ*(*x*, ·) is an Orlicz function, *φ* is said to be of* uniformly lower* (resp.,* upper*)* type* *p* with *p* ∈ (−*∞*, *∞*), if there exists a positive constant *C* such that, for all *x* ∈ ℝ^*n*^, *t* ∈ (0, *∞*), and *s* ∈ (0, 1) (resp., *s* ∈ [1, *∞*)),

(4) $$\begin{array}{c} \varphi \left(x,st\right)\leq Cs^{p}\varphi \left(x,t\right); \end{array}$$

*φ* is said to be of* positive uniformly lower *(resp.,* upper*)* type* if it is of uniformly lower (resp., upper) type *p* for some *p* ∈ (0, *∞*). Let

(5) i(φ)≔sup⁡{p∈(−∞,∞):      φ  is  of  uniformly  lower  type  p},I(φ)≔inf⁡{p∈(−∞,∞):     φ  is  of  uniformly  upper  type  p}

denote the* uniformly critical lower type* and the* critical upper type* of the function *φ*, respectively.

Now we recall the notion of expansive dilations on ℝ^*n*^; see [9]. A real *n* × *n* matrix *A* is called an* expansive dilation*, shortly a* dilation*, if min⁡_*λ*∈*σ*(*A*)_ | *λ* | >1, where *σ*(*A*) denotes the set of all* eigenvalues* of *A*. Let *λ*_−_ and *λ*_+_ be two* positive numbers* such that

(6) $$\begin{array}{c} 1<\lambda_{-}<\min\left\{\left|\lambda \right|:\lambda \in \sigma \left(A\right)\right\}\leq \max\left\{\left|\lambda \right|:\lambda \in \sigma \left(A\right)\right\}<\lambda_{+}. \end{array}$$

In the case when *A* is diagonalizable over *ℂ*, we can even take *λ*_−_≔min⁡{|*λ* | :*λ* ∈ *σ*(*A*)} and *λ*_+_≔max⁡{|*λ*| : *λ* ∈ *σ*(*A*)}. Otherwise, we need to choose them sufficiently close to these equalities according to what we need in our arguments.

It was proved in [9, Lemma 2.2] that, for a given dilation *A*, there exist an open ellipsoid Δ and *r* ∈ (1, *∞*) such that Δ ⊂ *r*Δ ⊂ *A*Δ, and one can additionally assume that |Δ | = 1, where |Δ| denotes the *n*-dimensional Lebesgue measure of the set Δ. Let *B*_*k*_≔*A* ^*k*^Δ for *k* ∈ *ℤ*. Then *B*_*k*_ is open, *B*_*k*_ ⊂ *rB*_*k*_ ⊂ *B*_*k*+1_, and |*B*_*k*_ | = *b* ^*k*^. Throughout the whole paper, let *σ* be the* minimal integer* such that *r* ^*σ*^ ≥ 2 and, for any subset *E* of ℝ^*n*^, let *E* ^∁^≔ℝ^*n*^∖*E*. Then, for all *k*, *j* ∈ *ℤ* with *k* ≤ *j*, it holds true that

(7) $$\begin{array}{c} B_{k}+B_{j}\subset B_{j+\sigma }, \end{array}$$

(8) $$\begin{array}{c} B_{k}+{\left(B_{k+\sigma }\right)}^{∁}\subset {\left(B_{k}\right)}^{∁}, \end{array}$$

where *E* + *F* denotes the algebraic sums {*x* + *y* : *x* ∈ *E*, *y* ∈ *F*} of sets *E*, *F* ⊂ ℝ^*n*^.

**Definition 1**

A* quasinorm*, associated with an expansive matrix *A*, is a Borel measurable mapping *ρ*_*A*_ : ℝ^*n*^ → [0, *∞*), for simplicity, denoted by *ρ*, such that

- *ρ*(*x*) > 0 for all *x* ∈ ℝ∖{0};
- *ρ*(*Ax*) = *bρ*(*x*) for all *x* ∈ ℝ^*n*^, where *b*≔|det⁡*A*|;
- *ρ*(*x* + *y*) ≤ *H*[*ρ*(*x*) + *ρ*(*y*)] for all *x*, *y* ∈ ℝ^*n*^, where *H* ∈ [1, *∞*) is a constant.

In the standard dyadic case *A*≔2*I*_*n*×*n*_, *ρ*(*x*)≔|*x*|^*n*^ for all *x* ∈ ℝ^*n*^ is an example of homogeneous quasinorms associated with *A*; here and hereafter, *I*_*n*×*n*_ always denotes the *n* × *n* *unit matrix* and |·| the Euclidean norm in ℝ^*n*^.

It was proved in [9, Lemma 2.4] that all homogeneous quasinorms associated with a given dilation *A* are equivalent. Therefore, for a given expansive dilation *A*, in what follows, for convenience, we always use the* step homogeneous quasinorm* *ρ* defined by setting, for all *x* ∈ ℝ^*n*^,

(9) $$\begin{array}{c} \rho \left(x\right)≔\underset{k\in \mathbb{Z}}{\sum }b^{k}\chi_{B_{k+1}\setminus B_{k}}\left(x\right)\;\text{if}\;\;x\neq 0,\;\;\text{or}\;\;\text{else}\;\;\rho \left(0\right)≔0. \end{array}$$

By (7) and (8), we know that, for all *x*, *y* ∈ ℝ^*n*^,

(10) $$\begin{array}{c} \rho \left(x+y\right)\leq b^{\sigma }\left(\max\left\{\rho \left(x\right),\rho \left(y\right)\right\}\right)\leq b^{\sigma }\left[\rho \left(x\right)+\rho \left(y\right)\right]; \end{array}$$

see [9, page 8]. Moreover, (ℝ^*n*^, *ρ*, *dx*) is a space of homogeneous type in the sense of Coifman and Weiss [41], where *dx* denotes the *n*-dimensional Lebesgue measure.

**Definition 2**

Let *p* ∈ [1, *∞*). A function *φ* : ℝ^*n*^ × [0, *∞*)→[0, *∞*) is said to satisfy the* uniform anisotropic Muckenhoupt condition* *𝔸*_*p*_(*A*), denoted by *φ* ∈ *𝔸*_*p*_(*A*), if there exists a positive constant *C* such that, for all *t* ∈ (0, *∞*), when *p* ∈ (1, *∞*),

(11) sup⁡x∈ℝnsup⁡k∈ℤ{b−k∫x+Bkφ(y,t)dy}  ×{b−k∫x+Bk[φ(y,t)]−1/(p−1)dy}p−1≤C

and, when *p* = 1,

(12) $$\begin{array}{c} \underset{x\in {\mathbb{R}}^{n}}{\sup}\underset{k\in \mathbb{Z}}{\sup}\left\{b^{-k}\int_{x+B_{k}}\varphi \left(y,t\right)dy\right\}\left\{\underset{y\in x+B_{k}}{\text{ess sup}}{\left[\varphi (y,t)\right]}^{-1}\right\}\leq C. \end{array}$$

The minimal constant *C* as above is denoted by *C*_*p*,*A*,*n*_(*φ*).

Define *𝔸*_*∞*_(*A*)≔⋃_1≤*p*<*∞*_ *𝔸*_*p*_(*A*) and

(13) $$\begin{array}{c} q\left(\varphi \right)≔\inf\left\{q\in \left[1,\infty \right):\varphi \in {𝔸}_{q}\left(A\right)\right\}. \end{array}$$

If *φ* ∈ *𝔸*_*∞*_(*A*) is independent of *t* ∈ [0, *∞*), then *φ* is just an anisotropic Muckenhoupt *A*_*∞*_(*A*) weight in [42]. Obviously, *q*(*φ*)∈[1, *∞*). If *q*(*φ*)∈(1, *∞*), by a discussion similar to [6, page 3072], it is easy to know *φ* ∉ *𝔸*_*q*(*φ*)_(*A*). Moreover, there exists a *φ* ∈ (∩_*q*>1_ *𝔸*_*q*_(*A*))∖*𝔸*_1_(*A*) such that *q*(*φ*) = 1; see Johnson and Neugebauer [43, page 254, Remark].

Now, we introduce anisotropic growth functions.

**Definition 3**

A function *φ* : ℝ^*n*^ × [0, *∞*)→[0, *∞*) is called an* anisotropic growth function* if

- the function *φ* is an anisotropic Musielak-Orlicz function; that is,
  - the function *φ*(*x*, ·):[0, *∞*)→[0, *∞*) is an Orlicz function for all *x* ∈ ℝ^*n*^,
  - the function *φ*(·, *t*) is a Lebesgue measurable function for all *t* ∈ [0, *∞*);
- the function *φ* belongs to *𝔸*_*∞*_(*A*);
- the function *φ* is of positive uniformly lower type *p* for some *p* ∈ (0, 1] and of uniformly upper type 1.

Given a growth function *φ*, let

(14) $$\begin{array}{c} m\left(\varphi \right)≔\left\lfloor \left(\frac{q\left(\varphi \right)}{i\left(\varphi \right)}-1\right)\frac{\ln b}{\ln\lambda_{-}}\right\rfloor . \end{array}$$

Clearly,

(15) $$\begin{array}{c} \varphi \left(x,t\right)≔w\left(x\right)\Phi \left(t\right)\;\forall x\in {\mathbb{R}}^{n},\;\;t\in \left[0,\infty \right) \end{array}$$

is an anisotropic growth function if *w* is a classical or an anisotropic *A*_*∞*_ Muckenhoupt weight (cf. [42]) and Φ of positive lower type *p* for some *p* ∈ (0, 1] and of upper type 1. More examples of growth functions can be found in [20, 22, 30, 32].

**Remark 4**

By Lemma 11 below (see also [20, Lemma 4.1]), without loss of generality, we may always assume that an anisotropic growth function *φ* is of positive uniformly lower type *p* for some *p* ∈ (0, 1] and of uniformly upper type 1 such that *φ*(*x*, ·) is continuous and strictly increasing for all given *x* ∈ ℝ^*n*^.

Throughout the whole paper, we always assume that *φ* is an anisotropic growth function. Recall that the* Musielak-Orlicz-type space* *L* ^*φ*^(ℝ^*n*^) is defined to be the set of all measurable functions *f* such that, for some *λ* ∈ (0, *∞*),

(16) $$\begin{array}{c} \int_{{\mathbb{R}}^{n}}\varphi \left(x,\frac{\left|f\left(x\right)\right|}{\lambda }\right)dx<\infty \end{array}$$

with the Luxembourg (or called the Luxembourg-Nakano) (quasi)norm

(17) $$\begin{array}{c} {||f||}_{L^{\varphi }({\mathbb{R}}^{n})}≔\inf\left\{\lambda \in \left(0,\infty \right):\int_{{\mathbb{R}}^{n}}\varphi \left(x,\frac{\left|f\left(x\right)\right|}{\lambda }\right)dx\leq 1\right\}. \end{array}$$

For *m* ∈ *ℕ*, let

(18) 𝒮m(ℝn)≔{ϕ∈𝒮(ℝn): sup⁡x∈ℝn,|α|≤m+1[1+ρ(x)]m+2|∂αϕ(x)|≤1}.

In what follows, for *φ* ∈ *𝒮*(ℝ^*n*^), *k* ∈ *ℤ*, and *x* ∈ ℝ^*n*^, let *φ*_*k*_(*x*)≔*b* ^*k*^ *φ*(*A* ^*k*^ *x*).

For *f* ∈ *𝒮*′(ℝ^*n*^), the* nontangential grand maximal function f*_m_* of *f* is defined by setting, for all *x* ∈ ℝ^*n*^,

(19) $$\begin{array}{c} f_{m}^{*}\left(x\right)≔\underset{\underset{k\in \mathbb{Z}}{\phi \in {𝒮}_{m}\left({\mathbb{R}}^{n}\right)}}{\sup}\underset{y\in x+B_{k}}{\sup}\left|f*\phi_{k}\left(y\right)\right|. \end{array}$$

If *m*≔*m*(*φ*), we then write f* instead of *f*_m_*.

**Definition 5**

For any *m* ∈ *ℕ* and anisotropic growth function *φ*, the* anisotropic Hardy space* *H*_*m*,*A*_^*φ*^(ℝ^*n*^) of Musielak-Orlicz type is defined to be the set of all *f* ∈ *𝒮*′(ℝ^*n*^) such that *f*_m_* ∈ *L* ^*φ*^(ℝ^*n*^) with the (quasi)norm ||*f*||_*H*_*m*,*A*_^*φ*^(ℝ^*n*^)_≔||*f*_m_*||_*L*^*φ*^(ℝ^*n*^)_. When *m*≔*m*(*φ*), *H*_*m*,*A*_^*φ*^(ℝ^*n*^) is denoted simply by *H*_*A*_^*φ*^(ℝ^*n*^).

Observe that, when *A*≔2*I*_*n*×*n*_ and *φ* is as in (15) with a Muckenhoupt weight *w* and an Orlicz function Φ, the above Hardy spaces *H*_*A*_^*φ*^(ℝ^*n*^) are just weighted Hardy-Orlicz spaces which include classical Hardy-Orlicz spaces of Janson [44] (*w* ≡ 1 in this context) and classical weighted Hardy spaces of García-Cuerva [8] as well as Strömberg and Torchinsky [5] (Φ(*t*)≔*t* ^*p*^ for all *t* ∈ [0, *∞*) in this context); see also [19, 45, 46]. When *φ* is as in (15) with Φ(*t*)≔*t* ^*p*^ for all *t* ∈ [0, *∞*), the above Hardy spaces *H*_*A*_^*φ*^(ℝ^*n*^) become weighted anisotropic Hardy spaces (see [6]) and, more generally, when Φ is an Orlicz function, these Hardy spaces are new.

Now let us give some basic properties of *H*_*m*,*A*_^*φ*^(ℝ^*n*^).

**Proposition 6**

For *m* ∈ *ℕ*, it holds true that *H*_*m*,*A*_^*φ*^(ℝ^*n*^) ⊂ *𝒮*′(ℝ^*n*^) and the inclusion is continuous.

**Proof**

Let *f* ∈ *H*_*m*,*A*_^*φ*^(ℝ^*n*^). For any *ϕ* ∈ *𝒮*(ℝ^*n*^) and *x* ∈ *B*_0_, we have 〈*f*, *ϕ*〉 = *f*∗*ψ*_*x*_(*x*), where *ψ*_*x*_(*y*)≔*ϕ*(*x* − *y*) for all *y* ∈ ℝ^*n*^.

By Definition 1, we see that

(20) $$\begin{array}{c} \underset{x\in B_{0},y\in {\mathbb{R}}^{n}}{\sup}\frac{1+\rho \left(y\right)}{1+\rho \left(x-y\right)}\leq b^{2\sigma }. \end{array}$$

Therefore, it holds true that

(21) |〈f,ϕ〉|=||ψx||𝒮m(ℝn)|f∗(ψx||ψx||𝒮m(ℝn))(x)|≤b2σ(m+2)||ϕ||𝒮m(ℝn)inf⁡x∈B0fm∗(x)≤b2σ(m+2)||ϕ||𝒮m(ℝn)||χB0||Lφ(ℝn)−1||f||Hm,Aφ(ℝn).

This implies that *f* ∈ *𝒮*′(ℝ^*n*^) and the inclusion is continuous, which completes the proof of Proposition 6.

Using Proposition 6, with an argument similar to that of [20, Proposition 5.2], we have the following conclusion, the details being omitted.

**Proposition**

Let *m* ∈ *ℕ* and let *φ* be an anisotropic growth function. Then *H*_*m*,*A*_^*φ*^(ℝ^*n*^) is complete.

## 3. Characterizations of *H*_*A*_^*φ*^(ℝ^*n*^) via Maximal Functions

The goal of this section is to establish some maximal function characterizations of *H*_*A*_^*φ*^(ℝ^*n*^). Let us begin with the notions of anisotropic variants of the radial, the nontangential, and the tangential maximal functions.

**Definition**

Let *ψ* ∈ *𝒮*(ℝ^*n*^) with ∫_ℝ^*n*^_ *ψ*(*x*)*dx* ≠ 0. The anisotropic radial, the nontangential, and the tangential maximal functions of *f* associated to *ψ* are defined, respectively, by setting, for all *x* ∈ ℝ^*n*^,

(22) $$\begin{array}{c} {ℳ}_{\psi }^{0}f\left(x\right)≔\underset{k\in \mathbb{Z}}{\sup}\left|\psi_{k}*f\left(x\right)\right|,\\ {ℳ}_{\psi }f\left(x\right)≔\underset{k\in \mathbb{Z}}{\sup}\underset{y\in x+B_{k}}{\sup}\left|\psi_{k}*f\left(y\right)\right|,\\ T_{\psi }^{N}f\left(x\right)≔\underset{k\in \mathbb{Z}}{\sup}\underset{y\in {\mathbb{R}}^{n}}{\sup}\frac{\left|\psi_{k}*f\left(y\right)\right|}{{\left[1+\rho \left(A^{-k}\left(x-y\right)\right)\right]}^{N}},\;N\in {\mathbb{Z}}_{+}. \end{array}$$

**Theorem 9**

Let *φ* be an anisotropic growth function and *ψ* ∈ *𝒮*(ℝ^*n*^) with ∫_ℝ^*n*^_ *ψ*(*x*)*dx* ≠ 0. Then, for any *f* ∈ *𝒮*′(ℝ^*n*^), the following are equivalent:

(23) $$\begin{array}{c} f\in H_{A}^{\varphi }\left({\mathbb{R}}^{n}\right); \end{array}$$

(24) $$\begin{array}{c} T_{\psi }^{N}f\in L^{\varphi }\left({\mathbb{R}}^{n}\right),\;N>\frac{{\left[q\left(\varphi \right)\right]}^{2}}{i\left(\varphi \right)}; \end{array}$$

(25) $$\begin{array}{c} {ℳ}_{\psi }f\in L^{\varphi }\left({\mathbb{R}}^{n}\right); \end{array}$$

(26) $$\begin{array}{c} {ℳ}_{\psi }^{0}f\in L^{\varphi }\left({\mathbb{R}}^{n}\right). \end{array}$$

Moreover, for sufficiently large *m*, there exist positive constants *C*_1_, *C*_2_, *C*_3_, and *C*_4_, independent of *f* ∈ *H*_*A*_^*φ*^(ℝ^*n*^), such that

(27) ||f||HAφ(ℝn)≔||fm∗||Lφ(ℝn)≤C1||ℳψf||Lφ(ℝn)≤C2||ℳψ0f||Lφ(ℝn)≤C3||TψNf||Lφ(ℝn)≤C4||f||HAφ(ℝn).

The approach we use to prove Theorem 9 is motivated by Bownik [9, Theorem 7.1]. First, we need the following two lemmas which come from [5, pages 7-8] and [20, Lemma 4.1(ii)].

In what follows, for any set *E* and *t* ∈ [0, *∞*), let

(28) $$\begin{array}{c} \varphi \left(E,t\right):=\int_{E}\varphi \left(x,t\right)dx. \end{array}$$

**Lemma 10**

Let *q* ∈ [1, *∞*) and *φ* ∈ *𝔸*_*q*_(*A*). Then there exists a positive constant *C* such that, for all *x* ∈ ℝ^*n*^, *k* ∈ *ℤ*, *E* ⊂ (*x* + *B*_*k*_), and *t* ∈ (0, *∞*),

(29) $$\begin{array}{c} \frac{\varphi \left(x+B_{k},t\right)}{\varphi \left(E,t\right)}\leq C\frac{{\left|x+B_{k}\right|}^{q}}{{\left|E\right|}^{q}}. \end{array}$$

**Lemma 11**

Let *φ* be an anisotropic growth function. For all (*x*, *t*) ∈ ℝ^*n*^ × [0, *∞*), $\tilde{\varphi }(x,t)≔\int_{0}^{t}(\varphi (x,s)/s)ds$ is also an anisotropic growth function which is equivalent to *φ*; moreover, $\tilde{\varphi }(x,\cdot )$ for any given *x* ∈ ℝ^*n*^ is continuous and strictly increasing.

We now recall some Peetre-type maximal functions from [9]. These maximal functions are obtained via the truncation with an additional extra decay term. Namely, for an integer *K* representing the truncation level and a real nonnegative number *L* representing the decay level, any *x* ∈ ℝ^*n*^ and *k* ∈ *ℤ*, we define

(30) $$\begin{array}{c} m_{K,L}\left(x,k\right)≔{\left[\max\left\{1,\rho \left(A^{-K}x\right)\right\}\right]}^{L}{\left(1+b^{-k-K}\right)}^{L} \end{array}$$

and the following Peetre-type radial, the nontangential, the tangential, the radial grand, and the nontangential grand maximal functions:

(31) ℳψ0(K,L)f(x)≔sup⁡k∈ℤk≤K|ψk∗f(x)|mK,L(x,k),ℳψ(K,L)f(x)≔sup⁡k∈ℤk≤Ksup⁡y∈x+Bk|ψk∗f(y)|mK,L(y,k),TψN(K,L)f(x)≔sup⁡k∈ℤk≤K sup⁡y∈ℝn|ψk∗f(y)|[1+ρ(A−k(x−y))]NmK,L(y,k),N∈ℤ+,fm0,∗(K,L)(x)≔sup⁡ψ∈𝒮m(ℝn)ℳψ0(K,L)f(x),fm∗(K,L)(x)≔sup⁡ψ∈𝒮m(ℝn)ℳψ(K,L)f(x),

where *𝒮*_*m*_(ℝ^*n*^) is as in (18).

We need some technical lemmas. To begin with, let *F* : ℝ^*n*^ × *ℤ* → [0, *∞*) be an arbitrary Borel measurable function. For fixed *j* ∈ *ℤ* and *K* ∈ *ℤ* ∪ {*∞*}, the* maximal function* of *F* with aperture *j* is defined by setting, for all *x* ∈ ℝ^*n*^,

(32) $$\begin{array}{c} F_{j}^{*,K}\left(x\right)≔\underset{\underset{k\leq K}{k\in \mathbb{Z}}\;}{\sup}\underset{y\in x+B_{j+k}}{\sup}F\left(y,k\right). \end{array}$$

It was shown in [9, page 42] that *F*_*j*_^∗,*K*^ is lower semicontinuous; namely, {*x* ∈ ℝ^*n*^ : *F*_*j*_^∗,*K*^(*x*) > *λ*} is open for any *λ* ∈ (0, *∞*).

We have the following Lemma 12 associated to *F*_*j*_^∗,*K*^ which is a uniformly weighted analogue of [9, Lemma 7.2].

**Lemma 12**

Let *q* ∈ [1, *∞*) and *φ* ∈ *𝔸*_*q*_(*A*). Then there exists a positive constant *C* such that, for any *λ*, *t* ∈ [0, *∞*) and *j* ∈ *ℤ*_+_,

(33) φ({x∈ℝn:Fj∗,K(x)>t},λ)  ≤Cbq2jφ({x∈ℝn:F0∗,K(x)>t},λ),

(34) $$\begin{array}{c} \int_{{\mathbb{R}}^{n}}\varphi \left(x,F_{j}^{*,K}\left(x\right)\right)dx\leq Cb^{q^{2}j}\int_{{\mathbb{R}}^{n}}\varphi \left(x,F_{0}^{*,K}\left(x\right)\right)dx. \end{array}$$

**Proof**

For any *t* ∈ [0, *∞*), let *Ω*≔{*x* ∈ ℝ^*n*^ : *F*_0_^∗,*K*^(*x*) > *t*}. For any *x* ∈ ℝ^*n*^ satisfying *F*_*j*_^∗,*K*^(*x*) > *t*, there exist *k* ≤ *K* and *y* ∈ *x* + *B*_*k*+*j*_ such that *F*(*y*, *k*) > *t*. Clearly, *y* + *B*_*k*_ ⊂ *Ω*. Moreover, by (7) and *j* ∈ *ℤ*_+_, we find that

(35) $$\begin{array}{c} y+B_{k}\subset x+B_{k+j}+B_{k}\subset x+B_{k+j+\sigma }. \end{array}$$

From this and *φ* ∈ *𝔸*_*q*_(*A*) with Lemma 10, it follows that

(36) $$\begin{array}{c} b^{-q(j+\sigma )}\varphi \left(x+B_{k+j+\sigma },\lambda \right)\leq C_{1}\varphi \left(y+B_{k},\lambda \right). \end{array}$$

Consequently, by this and *y* + *B*_*k*_ ⊂ *Ω*∩(*x* + *B*_*k*+*j*+*σ*_), we have

(37) φ(Ω∩(x+Bk+j+σ),λ)≥φ(y+Bk,λ)≥C1−1b−q(j+σ)×φ(x+Bk+j+σ,λ),

which implies that

(38) $$\begin{array}{c} {ℳ}_{\varphi (\cdot ,\lambda )}\left(\chi_{\Omega }\right)\left(x\right)\geq C_{1}^{-1}b^{-q(j+\sigma )}, \end{array}$$

where *ℳ*_*φ*(·,*λ*)_ denotes the* centered Hardy-Littlewood maximal function* associated to the measure *φ*(*x*, *λ*)*dx*; namely, for all *x* ∈ ℝ^*n*^,

(39) ℳφ(·,λ)f(x)≔sup⁡m∈ℤ1φ(x+Bm,λ)×∫x+Bm|f(y)|φ(y,λ)dy.

Thus,

(40) {x∈ℝn:Fj∗,K(x)>t} ⊂{x∈ℝn:ℳφ(·,λ)(χΩ)(x)≥C1−1b−q(j+σ)}.

From this and the weak-*L* ^*q*^(ℝ^*n*^, *φ*(*x*, *λ*)*dx*) boundedness of *ℳ*_*φ*(·,*λ*)_ with *φ* ∈ *𝔸*_*q*_(*A*), it is easy to deduce (33).

Next we prove (34). By Lemma 11, we know that

(41) ∫ℝnφ(x,Fj∗,K(x))dx~∫ℝn∫0Fj∗,K(x)φ(x,t)dttdx~∫0∞∫{x∈ℝn:Fj∗,K(x)>t}φ(x,t)dxdtt,

which, together with (33), further implies that

(42) ∫ℝnφ(x,Fj∗,K(x))dx ≲bq2j∫0∞∫{x∈ℝn:F0∗,K(x)>t}φ(x,t)dxdtt ~bq2j∫ℝnφ(x,F0∗,K(x))dx,

which is desired. This finishes the proof of Lemma 12.

The following Lemma 13 is just [20, Lemma 4.1(i)].

**Lemma 13**

Let *φ* be an anisotropic growth function. Then there exists a positive constant *C* such that, for all (*x*, *t*_*j*_) ∈ ℝ^*n*^ × [0, *∞*) with *j* ∈ *ℕ*,

(43) $$\begin{array}{c} \varphi \left(x,\overset{\infty }{\underset{j=1}{\sum }}t_{j}\right)\leq C\overset{\infty }{\underset{j=1}{\sum }}\varphi \left(x,t_{j}\right). \end{array}$$

The following Lemma 14 extends [9, Lemma 7.5] to the setting of anisotropic Musielak-Orlicz function spaces.

**Lemma 14**

Let *ψ* ∈ *𝒮*(ℝ^*n*^), let *φ* be an anisotropic growth function, and let *N* ∈ ([*q*(*φ*)]^2^/*i*(*φ*), *∞*). Then there exists a positive constant *C* such that, for all *K* ∈ *ℤ*, *L* ∈ [0, *∞*) and *f* ∈ *𝒮*′(ℝ^*n*^),

(44) ||TψN(K,L)f||Lφ(ℝn)≤C||ℳψ(K,L)f||Lφ(ℝn).

**Proof**

For any *f* ∈ *𝒮*′(ℝ^*n*^), *ψ* ∈ *𝒮*(ℝ^*n*^), *K* ∈ *ℤ*, and *L* ∈ [0, *∞*), consider a function *F* : ℝ^*n*^ × *ℤ* → [0, *∞*) given by setting, for all (*y*, *k*) ∈ ℝ^*n*^ × *ℤ*,

(45) $$\begin{array}{c} F\left(y,k\right)≔\frac{\left|f*\psi_{k}\left(y\right)\right|}{m_{K,L}\left(y,k\right)} \end{array}$$

with *m*_*K*,*L*_ being as in (30). Fix *x* ∈ ℝ^*n*^ and *N* ∈ ([*q*(*φ*)]^2^/*i*(*φ*), *∞*). If *k* ≤ *K* and *x* − *y* ∈ *B*_*k*_, then

(46) $$\begin{array}{c} F\left(y,k\right){\left[\max\left\{1,\rho \left(A^{-k}\left(x-y\right)\right)\right\}\right]}^{-N}\leq F_{0}^{*,K}\left(x\right), \end{array}$$

where *F*_0_^∗,*K*^ is as in (32). If *k* ≤ *K* and *x* − *y* ∈ *B*_*k*+*j*+1_∖*B*_*k*+*j*_ for some *j* ∈ *ℤ*_+_, then

(47) $$\begin{array}{c} F\left(y,k\right){\left[\max\left\{1,\rho \left(A^{-k}\left(x-y\right)\right)\right\}\right]}^{-N}\leq b^{-jN}F_{j}^{*,K}\left(x\right), \end{array}$$

where *F*_*j*_^∗,*K*^ is as in (32). By taking supremum over all *y* ∈ ℝ^*n*^ and *k* ≤ *K*, we obtain

(48) $$\begin{array}{c} T_{\psi }^{N(K,L)}f\left(x\right)\leq \overset{\infty }{\underset{j=0}{\sum }}b^{-jN}F_{j}^{*,K}\left(x\right). \end{array}$$

Moreover, since *N* ∈ ([*q*(*φ*)]^2^/*i*(*φ*), *∞*), we choose *p* < *i*(*φ*) large enough and *q* > *q*(*φ*) small enough such that *Np* − *q* ^2^ > 0. Therefore, from this, (48), Lemma 13, the uniformly lower type *p* of *φ*, and Lemma 12, it follows that

(49) ∫ℝnφ(x,TψN(K,L)f(x))dx  ≤∑j=0∞b−jNp∫ℝnφ(x,Fj∗,K(x))dx  ≲∑j=0∞b−j(Np−q2)∫ℝnφ(x,F0∗,K(x))dx  ≲∫ℝnφ(x,ℳψ(K,L)f(x))dx,

which implies (44). This finishes the proof of Lemma 14.

The following Lemmas 16 and 18 are just [9, Lemmas  7.5 and 7.6], respectively.

**Lemma 15**

Suppose *ψ* ∈ *𝒮*(ℝ^*n*^) with ∫_ℝ^*n*^_ *ψ*(*x*)*dx* ≠ 0. Then, for any given *N*, *L* ∈ [0, *∞*), there exist a positive integer *m* and a positive constant *C* such that, for all *f* ∈ *𝒮*′(ℝ^*n*^), integers *K* ∈ *ℤ*_+_ and *x* ∈ ℝ^*n*^,

(50) $$\begin{array}{c} f_{m}^{*,0(K,L)}\left(x\right)\leq CT_{\psi }^{N(K,L)}f\left(x\right). \end{array}$$

**Lemma 16**

Let *ψ* ∈ *𝒮*(ℝ^*n*^) with ∫_ℝ^*n*^_ *ψ*(*x*)*dx* ≠ 0 and *f* ∈ *𝒮*′(ℝ^*n*^). Then, for every *M* ∈ (0, *∞*), there exists *L* ∈ (0, *∞*) such that, for all *x* ∈ ℝ^*n*^,

(51) $$\begin{array}{c} {ℳ}_{\psi }^{\left(K,L\right)}f\left(x\right)\leq C{\left[\max\left\{1,\rho \left(x\right)\right\}\right]}^{-M}, \end{array}$$

where *C* is a positive constant depending on *K*, *M*, *L* ∈ *ℤ*_+_, *A*, and *ψ*, but independent of *f* and *x*.

The following Lemma 17 is just [9, Proposition 3.10] and [6, Proposition 2.11].

**Lemma 17**

There exists a positive constant *C* such that, for almost every *x* ∈ ℝ^*n*^, *m* ∈ *ℕ*, and *f* ∈ *L*_loc⁡_^1^(ℝ^*n*^)∩*𝒮*′(ℝ^*n*^),

(52) $$\begin{array}{c} f\left(x\right)\leq f_{m}^{*}\left(x\right)\leq Cf_{m}^{*,0}\left(x\right)\leq C{ℳ}_{A}f\left(x\right), \end{array}$$

where *f*_*m*_^∗,0^(*x*)≔sup⁡_*ϕ*∈*𝒮*_*m*_(ℝ^*n*^)_sup⁡_*k*∈*ℤ*_|*f*∗*ϕ*_*k*_(*x*)| for all *x* ∈ ℝ^*n*^ and *ℳ*_*A*_ denotes the anisotropic Hardy-Littlewood maximal operator defined by setting, for all *x* ∈ ℝ^*n*^,

(53) $$\begin{array}{c} {ℳ}_{A}f\left(x\right)≔\underset{x\in B,B\in ℬ}{\sup}\frac{1}{\left|B\right|}\int_{B}\left|f\left(y\right)\right|dy. \end{array}$$

The following lemma comes from [22, Corollary 2.8] with a slight modification, the details being omitted.

**Lemma 18**

Let *φ* be an anisotropic Musielak-Orlicz function with uniformly lower type *p*_*φ*_^−^ and uniformly upper type *p*_*φ*_^+^ satisfying *q*(*φ*) < *p*_*φ*_^−^ ≤ *p*_*φ*_^+^ < *∞*, where *q*(*φ*) is as in (13). Then the Hardy-Littlewood maximal operator *ℳ*_*A*_ is bounded on *L* ^*φ*^(ℝ^*n*^).

**Proof of Theorem 9**

Obviously, (23) ⇒ (25) ⇒ (26). Let *φ* be an anisotropic growth function and let *ψ* ∈ *𝒮*(ℝ^*n*^) satisfy ∫_ℝ^*n*^_ *ψ*(*x*)*dx* ≠ 0. By (50) of Lemma 15 with *L* = 0 and *N* ∈ ([*q*(*φ*)]^2^/*i*(*φ*), *∞*), we know that there exists a positive integer *m* such that, for all *f* ∈ *𝒮*′(ℝ^*n*^), *x* ∈ ℝ^*n*^, and integers *K* ∈ *ℤ*_+_,

(54) $$\begin{array}{c} f_{m}^{*,0(K,0)}\left(x\right)≲T_{\psi }^{N(K,0)}f\left(x\right). \end{array}$$

From this and Lemma 14, it follows that, for all *f* ∈ *𝒮*′(ℝ^*n*^) and *K* ∈ *ℤ*_+_,

(55) ||fm∗,0(K,0)||Lφ(ℝn)≲||TψN(K,0)f||Lφ(ℝn)≲||ℳψ(K,0)f||Lφ(ℝn).

As *K* → *∞*, by the monotone convergence theorem and the continuity of *φ*(*x*, ·) (see Lemma 11), we have

(56) $$\begin{array}{c} {||f_{m}^{*,0}||}_{L^{\varphi }({\mathbb{R}}^{n})}≲{||T_{\psi }^{N}f||}_{L^{\varphi }({\mathbb{R}}^{n})}≲{||{ℳ}_{\psi }f||}_{L^{\varphi }({\mathbb{R}}^{n})}, \end{array}$$

which, together with Lemma 17, implies that (25) ⇒ (24) ⇒ (23). It remains to prove (26) ⇒ (23).

Suppose *ℳ*_*ψ*_^0^ *f* ∈ *L* ^*φ*^(ℝ^*n*^). By Lemma 16, we find some *L* ∈ (0, *∞*) such that (51) holds true, which implies that *ℳ*_*ψ*_^(*K*,*L*)^ *f* ∈ *L* ^*φ*^(ℝ^*n*^) for all *K* ∈ *ℤ*_+_. By Lemmas 14 and 15, we find *m* ∈ *ℕ* such that

(57) ∫ℝnφ(x,fm∗,0(K,L)(x))dx  ≤C1∫ℝnφ(x,ℳψ(K,L)f(x))dx

with a positive constant *C*_1_ being independent of *K* ∈ *ℤ*_+_. For any given *K* ∈ *ℤ*_+_, let

(58) $$\begin{array}{c} \Omega_{K}≔\left\{x\in {\mathbb{R}}^{n}:f_{m}^{0,*(K,L)}\left(x\right)\leq C_{2}{ℳ}_{\psi }^{\left(K,L\right)}f\left(x\right)\right\}, \end{array}$$

where *C*_2_≔[2*C*_1_]^1/*p*^ with *p* ∈ (0, *i*(*φ*)). We claim that

(59) $$\begin{array}{c} \int_{{\mathbb{R}}^{n}}\varphi \left(x,{ℳ}_{\psi }^{\left(K,L\right)}f\left(x\right)\right)\leq 2\int_{\Omega_{K}}\varphi \left(x,{ℳ}_{\psi }^{\left(K,L\right)}f\left(x\right)\right)dx. \end{array}$$

Indeed, by (57), the uniformly lower type *p* of *φ* and *C*_2_^−*p*^ *C*_1_ = 1/2, we have

(60) ∫ΩK∁φ(x,ℳψ(K,L)(x))<C2−p∫ΩK∁φ(x,fm0,∗(K,L)(x))dx≤C2−pC1∫ℝnφ(x,ℳψ(K,L)(x))dx.

Moreover, for any *x* ∈ *Ω*_*K*_ and *p* ∈ (0, *i*(*φ*)), we choose *q* ∈ (0, *p*) small enough such that 1/*q* > *q*(*φ*), where *q*(*φ*) is as in (13), and, by [9, page 48, (7.16)], we know that there exists a constant *C*_3_ ∈ (1, *∞*) such that, for all integers *K* ∈ *ℤ*_+_ and *x* ∈ *Ω*_*K*_,

(61) $$\begin{array}{c} {ℳ}_{\psi }^{\left(K,L\right)}f\left(x\right)\leq C_{3}{\left[{ℳ}_{A}\left({\left[{ℳ}_{\psi }^{0(K,L)}f\right]}^{q}\right)\left(x\right)\right]}^{1/q}. \end{array}$$

Furthermore, from the fact that *φ* is of uniformly upper type 1 and positive lower type *p* with *p* < *i*(*φ*), it follows that $\tilde{\varphi }(x,t)≔\varphi (x,t^{1/q})$ is of uniformly upper 1/*q* and lower type *p*/*q*. Consequently, using (59), (61), and Lemma 18 with $\tilde{\varphi }$, we obtain

(62) ∫ℝnφ(x,ℳψ(K,L)f(x))dx  ≤2∫ΩKφ(x,ℳψ(K,L)f(x))dx  ≤2C3∫ΩKφ(x,[ℳA([ℳψ0(K,L)f]q)(x)]1/q)dx  ≤C4∫ℝnφ(x,ℳψ0(K,L)f(x))dx,

where *C*_4_ depends on *L* ∈ [0, *∞*) but is independent of *K* ∈ *ℤ*_+_. This inequality is crucial, since it gives a bound of the nontangential maximal function by the radial maximal function in *L* ^*φ*^(ℝ^*n*^).

Since *ℳ*_*ψ*_^(*K*,*L*)^ *f*(*x*) converges pointwise and monotonically to *ℳ*_*ψ*_ *f*(*x*) for all *x* ∈ ℝ^*n*^ as *K* → *∞*, it follows that *ℳ*_*ψ*_ *f* ∈ *L* ^*φ*^(ℝ^*n*^) by (62), the continuity of *φ*(*x*, ·) (see Lemma 11), and the monotone convergence theorem. Therefore, by choosing *L* = 0 and using (62), the continuity of *φ*(*x*, ·), and the monotone convergence theorem, we conclude that ||*ℳ*_*ψ*_ *f*||_*L*^*φ*^(ℝ^*n*^)_ ≤ *C*_4_||*ℳ*_*ψ*_^0^ *f*||_*L*^*φ*^(ℝ^*n*^)_, where now the positive constant *C*_4_ corresponds to *L* = 0 and is independent of *f* ∈ *𝒮*′(ℝ^*n*^). Combining this, (56), and Lemma 17, we obtain the desired conclusion and hence complete the proof of Theorem 9.

## 4. Calderón-Zygmund Decompositions

In this section, by using the Calderón-Zygmund decomposition associated with grand maximal functions on anisotropic ℝ^*n*^ established in [6], we obtain some bounded estimates on *H*_*A*_^*φ*^(ℝ^*n*^). We follow the constructions in [2, 6].

Throughout this section we consider a tempered distribution *f* so that, for all *λ*, *t* ∈ (0, *∞*),

(63) $$\begin{array}{c} \int_{\left\{x\in {\mathbb{R}}^{n}:f_{m}^{*}(x)>\lambda \right\}}\varphi \left(x,t\right)dx<\infty , \end{array}$$

where *m* ≥ *m*(*φ*) is some fixed integer. For a given *λ* ∈ (0, *∞*), let

(64) $$\begin{array}{c} \Omega ≔\left\{x\in {\mathbb{R}}^{n}:f_{m}^{*}\left(x\right)>\lambda \right\}. \end{array}$$

By referring to [6, page 3081], we know that there exist a positive constant *L*, independent of *Ω* and *f*, a sequence {*x*_*j*_}_*j*_ ⊂ *Ω*, and a sequence of integers, {*ℓ*_*j*_}_*j*_, such that

(65) $$\begin{array}{c} \Omega =\cup_{j}\left(x_{j}+B_{\ell_{j}}\right), \end{array}$$

(66) $$\begin{array}{c} \left(x_{i}+B_{\ell_{i}-2\sigma }\right)\cap \left(x_{j}+B_{\ell_{j}-2\sigma }\right)=\emptyset \;\forall i,j\;\;\text{with}\;\;i\neq j, \end{array}$$

(67) $$\begin{array}{c} \left(x_{j}+B_{\ell_{j}+4\sigma }\right)\cap \Omega^{∁}=\emptyset ,\;\left(x_{j}+B_{\ell_{j}+4\sigma +1}\right)\cap \Omega^{∁}\neq \emptyset \;\forall j, \end{array}$$

(68) (xi+Bℓi+2σ)∩(xj+Bℓj+2σ)≠∅  implies  that|ℓi−ℓj|≤σ,

(69) $$\begin{array}{c} \#\left\{j:\left(x_{i}+B_{\ell_{i}+2\sigma }\right)\cap \left(x_{j}+B_{\ell_{j}+2\sigma }\right)\neq \emptyset \right\}\leq L\;\forall i. \end{array}$$

Here and hereafter, for a set *E*, #*E* denotes its cardinality.

Fix *θ* ∈ *𝒮*(ℝ^*n*^) such that supp⁡*θ* ⊂ *B*_*σ*_, 0 ≤ *θ* ≤ 1, and *θ* ≡ 1 on *B*_0_. For each *j* and all *x* ∈ ℝ^*n*^, define *θ*_*j*_(*x*)≔*θ*(*A* ^−*ℓ*_*j*_^(*x* − *x*_*j*_)). Clearly, supp⁡*θ*_*j*_ ⊂ *x*_*j*_ + *B*_*ℓ*_*j*_+*σ*_ and *θ*_*j*_ ≡ 1 on *x*_*j*_ + *B*_*ℓ*_*j*__. By (65) and (69), for any *x* ∈ *Ω*, we have 1 ≤ ∑_*j*_ *θ*_*j*_(*x*) ≤ *L*. For every *i* and all *x* ∈ ℝ^*n*^, define

(70) $$\begin{array}{c} \zeta_{i}\left(x\right)≔\frac{\theta_{i}\left(x\right)}{\sum_{j}\theta_{j}\left(x\right)}. \end{array}$$

Then *ζ*_*i*_ ∈ *𝒮*(ℝ^*n*^), supp⁡*ζ*_*i*_ ⊂ *x*_*i*_ + *B*_*ℓ*_*i*_+*σ*_, 0 ≤ *ζ*_*i*_ ≤ 1, *ζ*_*i*_ ≡ 1 on *x*_*i*_ + *B*_*ℓ*_*i*_−2*σ*_ by (66), and ∑_*i*_ *ζ*_*i*_ = *χ*_*Ω*_. Therefore, the family {*ζ*_*i*_}_*i*_ forms a smooth partition of unity on *Ω*.

Let *s* ∈ *ℤ*_+_ be some fixed integer and let *𝒫*_*s*_(ℝ^*n*^) denote the linear space of polynomials of degrees not more than *s*. For each *i* and *P* ∈ *𝒫*_*s*_(ℝ^*n*^), let

(71) $$\begin{array}{c} {||P||}_{i}≔{\left[\frac{1}{\int_{{\mathbb{R}}^{n}}\zeta_{i}\left(x\right)dx}\int_{{\mathbb{R}}^{n}}{\left|P\left(x\right)\right|}^{2}\zeta_{i}\left(x\right)dx\right]}^{1/2}. \end{array}$$

Then (*𝒫*_*s*_(ℝ^*n*^), ||·||_*i*_) is a finite dimensional Hilbert space. Let *f* ∈ *𝒮*′(ℝ^*n*^). For each *i*, since *f* induces a linear functional on *𝒫*_*s*_(ℝ^*n*^) via *Q* ↦ (1/∫_ℝ^*n*^_ *ζ*_*i*_(*x*)*dx*)〈*f*, *Qζ*_*i*_〉, by the Riesz lemma, we know that there exists a unique polynomial *P*_*i*_ ∈ *𝒫*_*s*_(ℝ^*n*^) such that, for all *Q* ∈ *𝒫*_*s*_(ℝ^*n*^),

(72) 1∫ℝnζi(x)dx〈f,Qζi〉=1∫ℝnζi(x)dx〈Pi,Qζi〉=1∫ℝnζi(x)dx∫ℝnPi(x)Q(x)ζi(x)dx.

For every *i*, define a distribution *b*_*i*_≔(*f* − *P*_*i*_)*ζ*_*i*_.

We will show that, for suitable choices of *s* and *m*, the series ∑_*i*_ *b*_*i*_ converges in *𝒮*′(ℝ^*n*^) and, in this case, we define *g*≔*f* − ∑_*i*_ *b*_*i*_ in *𝒮*′(ℝ^*n*^).

**Definition 19**

The representation *f* = *g* + ∑_*i*_ *b*_*i*_, where *g* and *b*_*i*_ are as above, is called a* Calderón-Zygmund decomposition* of degree *s* and height *λ* associated with *f*_m_*.

The remainder of this section consists of a series of lemmas. In Lemmas 20 and 21, we give some properties of the smooth partition of unity {*ζ*_*i*_}_*i*_. In Lemmas 22 through 25, we derive some estimates for the bad parts {*b*_*i*_}_*i*_. Lemmas 26 and 27 give some estimates over the good part *g*. Finally, Corollary 28 shows the density of *L*_*φ*(·,1)_^*q*^(ℝ^*n*^)∩*H*_*A*_^*φ*^(ℝ^*n*^) in *H*_*A*_^*φ*^(ℝ^*n*^), where *q* ∈ (*q*(*φ*), *∞*).

Lemmas 20 through 23 are essentially Lemmas 4.3 through 4.6 of [9], the details being omitted.

**Lemma**

There exists a positive constant *C*_1_, depending only on *m*, such that, for all *i* and *ℓ* ≤ *ℓ*_*i*_,

(73) $$\begin{array}{c} \underset{|\alpha |\leq m\;}{\sup}\underset{x\in {\mathbb{R}}^{n}}{\sup}\left|\partial^{\alpha }\left[\zeta_{i}\left(A^{\ell }\cdot \right)\right]\left(x\right)\right|\leq C_{1}. \end{array}$$

**Lemma 21**

There exists a positive constant *C*_2_, independent of *f* and *λ*, such that, for all *i*,

(74) $$\begin{array}{c} \underset{y\in {\mathbb{R}}^{n}}{\sup}\left|P_{i}\left(y\right)\zeta_{i}\left(y\right)\right|\leq C_{2}\underset{y\in \left(x_{i}+B_{\ell_{i}+4\sigma +1}\right)\cap \Omega^{∁}}{\sup}f_{m}^{*}\left(y\right)\leq C_{2}\lambda . \end{array}$$

**Lemma 22**

There exists a positive constant *C*_3_, independent of *f* and *λ*, such that, for all *i* and *x* ∈ *x*_*i*_ + *B*_*ℓ*_*i*_+2*σ*_, (*b*_*i*_)_m_*(*x*) ≤ *C*_3_ *f*_m_*(*x*).

**Lemma 23**

If *m* ≥ *s* ≥ 0, then there exists a positive constant *C*_4_, independent of *f* and *λ*, such that, for all *t* ∈ *ℤ*_+_, *i*, and *x* ∈ *x*_*i*_ + *B*_*t*+*ℓ*_*i*_+2*σ*+1_∖*B*_*t*+*ℓ*_*i*_+2*σ*_, (*b*_*i*_)_m_*(*x*) ≤ *C*_4_ *λ*(*λ*_−_)^−*t*(*s*+1)^.

**Lemma 24**

If *m* ≥ *s* ≥ ⌊*q*(*φ*)ln⁡*b*/(*i*(*φ*)ln⁡*λ*_−_)⌋, then there exists a positive constant *C*_5_ such that, for all *f* ∈ *H*_*m*,*A*_^*φ*^(ℝ^*n*^), *λ* ∈ (0, *∞*), and *i*,

(75) $$\begin{array}{c} \int_{{\mathbb{R}}^{n}}\varphi \left(x,{\left(b_{i}\right)}_{m}^{*}\left(x\right)\right)dx\leq C_{5}\int_{x_{i}+B_{\ell_{i}+2\sigma }}\varphi \left(x,f_{m}^{*}\left(x\right)\right)dx. \end{array}$$

Moreover, the series ∑_*i*_ *b*_*i*_ converges in *H*_*m*,*A*_^*φ*^(ℝ^*n*^) and

(76) $$\begin{array}{c} \int_{{\mathbb{R}}^{n}}\varphi \left(x,{\left(\underset{i}{\sum }b_{i}\right)}_{m}^{*}\left(x\right)\right)dx\leq LC_{5}\int_{\Omega }\varphi \left(x,f_{m}^{*}\left(x\right)\right)dx, \end{array}$$

where *L* is as in (69).

**Proof**

By Lemma 22, we know that

(77) ∫ℝnφ(x,(bi)m∗(x))dx≲∫xi+Bℓi+2σφ(x,fm∗(x))dx+∫(xi+Bℓi+2σ)∁φ(x,(bi)m∗(x))dx.

Notice that *s* ≥ ⌊*q*(*φ*)ln⁡*b*/(*i*(*φ*)ln⁡*λ*_−_)⌋ implies that *b* ^−(*q*(*φ*)+*η*)^(*λ*_−_)^(*s*+1)*p*^ > 1 for sufficient small *η* > 0 and sufficient large *p* < *i*(*φ*). Using Lemma 10 with *φ* ∈ *𝔸*_*q*(*φ*)+*η*_(*A*), Lemma 23, and the fact that *f*_m_*(*x*) > *λ* for all *x* ∈ *x*_*i*_ + *B*_*ℓ*_*i*_+2*σ*_, we have

(78) ∫(xi+Bℓi+2σ)∁φ(x,(bi)m∗(x))dx =∑t=0∞∫xi+(Bt+ℓi+2σ+1∖Bt+ℓi+2σ)φ(x,(bi)m∗(x))dx ≲φ(xi+Bℓi+2σ,λ)∑t=0∞{b−[q(φ)+η](λ−)(s+1)p}−t ≲∫xi+Bℓi+2σφ(x,fm∗(x))dx,

which gives (75).

By (75) and (69), we see that

(79) ∫ℝn∑iφ(x,(bi)m∗(x))dx≲∑i∫xi+Bℓi+2σφ(x,fm∗(x))dx≲∫Ωφ(x,fm∗(x))dx,

which, together with the completeness of *H*_*m*,*A*_^*φ*^(ℝ^*n*^) (see Proposition 7), implies that ∑_*i*_ *b*_*i*_ converges in *H*_*m*,*A*_^*φ*^(ℝ^*n*^). So, by Proposition 6, we know that the series ∑_*i*_ *b*_*i*_ converges in *𝒮*′(ℝ^*n*^) and therefore (∑_*i*_ *b*_*i*_)_m_* ≤ ∑_*i*_(*b*_*i*_)_m_*. From this and Lemma 13, we deduce (76). This finishes the proof of Lemma 24.

Let *q* ∈ [1, *∞*]. We denote by *L*_*φ*(·,1)_^*q*^(ℝ^*n*^) the usually anisotropic weighted Lebesgue space with the anisotropic Muckenhoupt weight *φ*(·, 1). Then we have the following technical lemma (see [6, Lemma 4.8]), the details being omitted.

**Lemma 25**

If *q* ∈ (*q*(*φ*), *∞*] and *f* ∈ *L*_*φ*(·,1)_^*q*^(ℝ^*n*^), then the series ∑_*i*_ *b*_*i*_ converges in *L*_*φ*(·,1)_^*q*^(ℝ^*n*^), and there exists a positive constant *C*_6_, independent of *f* and *λ*, such that ||∑_*i*_|*b*_*i*_|||_*L*_*φ*(·,1)_^*q*^(ℝ^*n*^)_ ≤ *C*_6_||*f*||_*L*_*φ*(·,1)_^*q*^(ℝ^*n*^)_.

The following conclusion is essentially [9, Lemma 4.9], the details being omitted.

**Lemma 26**

If *m* ≥ *s* ≥ 0 and ∑_*i*_ *b*_*i*_ converges in *𝒮*′(ℝ^*n*^), then there exists a positive constant *C*_7_, independent of *f* and *λ*, such that, for all *x* ∈ ℝ^*n*^,

(80) $$\begin{array}{c} g_{m}^{*}\left(x\right)\leq C_{7}\lambda \underset{i}{\sum }{\left(\lambda_{-}\right)}^{-t_{i}(x)(s+1)}+f_{m}^{*}\left(x\right)\chi_{\Omega^{∁}}\left(x\right), \end{array}$$

where

(81) $$\begin{array}{c} t_{i}\left(x\right)≔\{\begin{array}{cc} \kappa_{i}, & if\;\;x\in x_{i}+\left(B_{\kappa_{i}+\ell_{i}+2\sigma +1}\setminus B_{\kappa_{i}+\ell_{i}+2\sigma }\right)\\ & for\;\;some\;\;\kappa_{i}\geq 0,\\ 0, & otherwise. \end{array} \end{array}$$

**Lemma 27**

Let *p* ∈ (*i*(*φ*), 1] and *q* ∈ (*q*(*φ*), *∞*).

(i) If *m* ≥ *s* ≥ ⌊*q*(*φ*)ln⁡*b*/(*i*(*φ*)ln⁡*λ*_−_)⌋ and *f* ∈ *H*_*m*,*A*_^*φ*^(ℝ^*n*^), then *g*_m_* ∈ *L*_*φ*(·,1)_^*q*^(ℝ^*n*^), and there exists a positive constant *C*_8_, independent of *f* and *λ*, such that
  (82) ∫ℝn[gm∗(x)]qφ(x,1)dx  ≤C8λq(max⁡{1λ,1λp})∫ℝnφ(x,fm∗(x))dx.
(ii) If *m* ∈ *ℕ* and *f* ∈ *L*_*φ*(·,1)_^*q*^(ℝ^*n*^), then *g* ∈ *L* ^*∞*^(ℝ^*n*^), and there exists a positive constant *C*_9_, independent of *f* and *λ*, such that ||*g*||_*L*^*∞*^(ℝ^*n*^)_ ≤ *C*_9_ *λ*.

**Proof**

Since *f* ∈ *H*_*m*,*A*_^*φ*^(ℝ^*n*^), by Lemma 24, we know that ∑_*i*_ *b*_*i*_ converges in *H*_*m*,*A*_^*φ*^(ℝ^*n*^) and therefore in *𝒮*′(ℝ^*n*^) by Proposition 6. Then, by Lemma 26, we have

(83) ∫ℝn[gm∗(x)]qφ(x,1)dx ≲λq∫ℝn[∑i(λ−)−ti(x)(s+1)]qφ(x,1)dx  +∫Ω∁[fm∗(x)]qφ(x,1)dx,

where *t*_*i*_(*x*) is as in Lemma 26. Observe that *m* ≥ ⌊*q*(*φ*)ln⁡*b*/(*i*(*φ*)ln⁡*λ*_−_)⌋ implies that (*λ*_−_)^*m*+1^ > *b* ^*q*(*φ*)^. Moreover, for any fixed *x* ∈ *x*_*i*_ + (*B*_*t*+*ℓ*_*i*_+2*σ*+1_∖*B*_*t*+*ℓ*_*i*_+2*σ*_) with *t* ∈ *ℤ*_+_, we find that

(84) b−t≲1|xi+Bt+ℓi+2σ+1|∫xi+Bt+ℓi+2σ+1χxi+Bℓi(y)dy≲ℳA(χxi+Bℓi)(x).

From this, the *L*_*φ*(·,1)_^*qq*(*φ*)^(*ℓ* ^*q*(*φ*)^)-boundedness of the vector-valued maximal function *ℳ*_*A*_ (see [42, Theorem 2.5]), (65), and (69), it follows that

(85) ∫ℝn[∑i(λ−)−ti(x)(m+1)]qφ(x,1)dx ≤∫ℝn[∑ib−ti(x)q(φ)]qφ(x,1)dx ≲∫ℝn{(∑i[ℳA(χxi+Bℓi)(x)]q(φ))1/q(φ)}qq(φ)   ×φ(x,1)dx ≲∫ℝn[∑i(χxi+Bℓi)q(φ)]qφ(x,1)dx ≲∫Ωφ(x,1)dx

and hence

(86) ∫ℝn[gm∗(x)]qφ(x,1)dx≲λq∫Ωφ(x,1)dx +∫Ω∁[fm∗(x)]qφ(x,1)dx.

Noticing that *f*_m_* > *λ* on *Ω*, then, for some *p* ∈ (0, *i*(*φ*)), we find that

(87) $$\begin{array}{c} \int_{\Omega }\varphi \left(x,1\right)dx≲\left(\max\left\{\frac{1}{\lambda },\frac{1}{\lambda^{p}}\right\}\right)\int_{\Omega }\varphi \left(x,f_{m}^{*}\left(x\right)\right)dx. \end{array}$$

On the other hand, since *f*_m_* ≤ *λ* on *Ω* ^∁^, for any *x* ∈ *Ω* ^∁^, using

(88) $$\begin{array}{c} \varphi \left(x,\lambda \right)≲\varphi \left(x,f_{m}^{*}\left(x\right)\right)\frac{\lambda^{q}}{{\left[f_{m}^{*}\left(x\right)\right]}^{q}}, \end{array}$$

we see that

(89) ∫Ω∁[fm∗(x)]qφ(x,1)dx  ≲(max⁡{1λ,1λp})∫Ω∁[fm∗(x)]qφ(x,λ)dx  ≲λq(max⁡{1λ,1λp})∫Ω∁φ(x,fm∗(x))dx.

Combining the above two estimates with (86), we obtain the desired conclusion of Lemma 27(i).

Moreover, notice that, if *f* ∈ *L*_*φ*(·,1)_^*q*^(ℝ^*n*^), then *g* and {*b*_*i*_}_*i*_ are functions. By Lemma 25, ∑_*i*_ *b*_*i*_ converges in *L*_*φ*(·,1)_^*q*^(ℝ^*n*^) and hence in *𝒮*′(ℝ^*n*^) due to the fact that *L*_*φ*(·,1)_^*q*^(ℝ^*n*^) ⊂ *𝒮*′(ℝ^*n*^) is continuous embedding (see [6, Lemma 2.8]). Write

(90) g=f−∑ibi=f(1−∑iζi)+∑iPiζi=fχΩ∁+∑iPiζi.

By Lemma 21 and (69), we have |*g*(*x*)|≲*λ* for all *x* ∈ *Ω*, and |*g*(*x*)| = |*f*(*x*)|≤*f*_m_*(*x*) ≤ *λ* for almost every *x* ∈ *Ω* ^∁^, which leads to ||*g*||_*L*^*∞*^(ℝ^*n*^)_≲*λ* and hence (ii) holds true. This finishes the proof of Lemma 27.

**Corollary 28**

For any *q* ∈ (*q*(*φ*), *∞*) and *m* ≥ ⌊*q*(*φ*)ln⁡*b*/(*i*(*φ*)ln⁡*λ*_−_)⌋, the subset *H*_*m*,*A*_^*φ*^(ℝ^*n*^)∩*L*_*φ*(·,1)_^*q*^(ℝ^*n*^) is dense in *H*_*m*,*A*_^*φ*^(ℝ^*n*^).

**Proof**

Let *f* ∈ *H*_*m*,*A*_^*φ*^(ℝ^*n*^). For any *λ* ∈ (0, *∞*), let *f* = *g* ^*λ*^ + ∑_*i*_ *b*_*i*_^*λ*^ be the Calderón-Zygmund decomposition of *f* of degree *s* with ⌊*q*(*φ*)ln⁡*b*/[*p*ln⁡(*λ*_−_)]⌋ ≤ *s* ≤ *m* and height *λ* associated with *f*_m_* as in Definition 19. Here, we rewrite *g* and *b*_*i*_ in Definition 19 into *g* ^*λ*^ and *b*_*i*_^*λ*^, respectively. By (76) of Lemma 24, we know that

(91) $$\begin{array}{c} {||\underset{i}{\sum }b_{i}^{\lambda }||}_{H_{m,A}^{\varphi }({\mathbb{R}}^{n})}≲\overset{}{\underset{\left\{x\in {\mathbb{R}}^{n}:f_{m}^{*}\left(x\right)>\lambda \right\}}{\int }}\varphi \left(x,f_{m}^{*}\left(x\right)\right)dx⟶0, \end{array}$$

and therefore *g* ^*λ*^ → *f* in *H*_*m*,*A*_^*φ*^(ℝ^*n*^) as *λ* → *∞*. Moreover, by Lemma 27(i), we see that (*g*_m_*)^*λ*^ ∈ *L*_*φ*(·,1)_^*q*^(ℝ^*n*^), which, together with Lemma 17, implies that *g* ^*λ*^ ∈ *L*_*φ*(·,1)_^*q*^(ℝ^*n*^). This finishes the proof of Corollary 28.

## 5. Atomic Characterizations of *H*_*A*_^*φ*^(ℝ^*n*^)

In this section, we establish the equivalence between *H*_*A*_^*φ*^(ℝ^*n*^) and anisotropic atomic Hardy spaces of Musielak-Orlicz type *H*_*A*_^*φ*,*q*,*s*^(ℝ^*n*^) (see Theorem 40 below).

Let *ℬ*≔{*B* = *x* + *B*_*k*_ : *x* ∈ ℝ^*n*^, *k* ∈ *ℤ*} be the collection of all dilated balls.

**Definition 29**

For any *B* ∈ *ℬ* and *q* ∈ [1, *∞*], let *L*_*φ*_^*q*^(*B*) be the set of all measurable functions *f*, supported in *B*, such that

(92) $$\begin{array}{c} {||f||}_{L_{\varphi }^{q}(B)}≔\{\begin{array}{cc} \underset{t\in (0,\infty )}{\sup}{\left[\frac{1}{\varphi \left(B,t\right)}\overset{}{\underset{{\mathbb{R}}^{n}}{\int }}{\left|f\left(x\right)\right|}^{q}\varphi \left(x,t\right)dx\right]}^{1/q}<\infty , & \\ q\in \left[1,\infty \right), & \\ {||f||}_{L^{\infty }(B)}<\infty ,q=\infty . & \end{array} \end{array}$$

It is easy to show that (*L*_*φ*_^*q*^(*B*), ||·||_*L*_*φ*_^*q*^(*B*)_) is a Banach space. Next we introduce anisotropic atomic Hardy spaces of Musielak-Orlicz type.

**Definition 30**

We have the following definitions.

(i) An anisotropic triplet (*φ*, *q*, *s*) is said to be* admissible*, if *q* ∈ (*q*(*φ*), *∞*] and *s* ∈ *ℤ*_+_ such that *s* ≥ *m*(*φ*) with *m*(*φ*) as in (14).
(ii) For an admissible anisotropic triplet (*φ*, *q*, *s*), a measurable function *a* is called an* anisotropic*  (*φ*, *q*, *s*)-*atom* if
  (a) *a* ∈ *L*_*φ*_^*q*^(*B*) for some *B* ∈ *ℬ*;
  (b) ||*a*||_*L*_*φ*_^*q*^(*B*)_ ≤ ||*χ*_*B*_||_*L*^*φ*^(ℝ^*n*^)_^−1^;
  (c) ∫_ℝ^*n*^_ *a*(*x*)*x* ^*α*^ *dx* = 0 for any |*α* | ≤*s*.
(iii) For an admissible anisotropic triplet (*φ*, *q*, *s*), the* anisotropic atomic Hardy space of Musielak-Orlicz type*, *H*_*A*_^*φ*,*q*,*s*^(ℝ^*n*^), is defined to be the set of all distributions *f* ∈ *𝒮*′(ℝ^*n*^) which can be represented as a sum of multiples of anisotropic (*φ*, *q*, *s*)-atoms, that is, *f* = ∑_*j*_ *a*_*j*_ in *𝒮*′(ℝ^*n*^), where *a*_*j*_ for *j* is a multiple of an anisotropic (*φ*, *q*, *s*)-atom supported in the dilated ball *x*_*j*_ + *B*_*ℓ*_*j*__, with the property
  (93) $$\begin{array}{c} \underset{j}{\sum }\varphi \left(x_{j}+B_{\ell_{j}},{||a_{j}||}_{L_{\varphi }^{q}(x_{j}+B_{\ell_{j}})}\right)<\infty . \end{array}$$
 Define
  (94) Λq({aj})≔inf⁡{λ∈(0,∞):∑jφ(xj+Bℓj,||aj||Lφq(xj+Bℓj)λ)≤1},||f||HAφ,q,s(ℝn)≔inf⁡{Λq({aj}):f=∑jaj  in  𝒮′(ℝn)},
 where the infimum is taken over all admissible decompositions of *f* as above.

**Remark 31**

(i) In Definition 30, if we assume that *f* can be represented as *f* = ∑_*j*_ *λ*_*j*_ *a*_*j*_ in *𝒮*′(ℝ^*n*^), where {*a*_*j*_}_*j*_ are (*φ*, *q*, *s*)-atoms supported in dilated balls {*x*_*j*_ + *B*_*ℓ*_*j*__}_*j*_, and

(95) ||f||H~Aφ,q,s(ℝn) ≔inf⁡{Λ~q({λj}):f=∑jλjaj  in  𝒮′(ℝn)}<∞,

where the infimum is taken over all admissible decompositions of *f* as above with

(96) Λ~q({λj}j)  ≔inf⁡{λ∈(0,∞):     ∑jφ(xj+Bℓj,|λj|||χxj+Bℓj||Lφ(ℝn)−1λ)≤1},

then the induced space ${\tilde{H}}_{A}^{\varphi ,q,s}({\mathbb{R}}^{n})$ and the space *H*_*A*_^*φ*,*q*,*s*^(ℝ^*n*^) coincide with equivalent (quasi)norms.

Indeed, if *f* = ∑_*j*_ *λ*_*j*_ *a*_*j*_ in *𝒮*′(ℝ^*n*^) for some (*φ*, *q*, *s*)-atoms, {*a*_*j*_}_*j*_, and {*λ*_*j*_}_*j*_ ⊂ *ℂ* such that ${\tilde{\Lambda }}_{q}(\{\lambda_{j}\})<\infty$. Write ${\tilde{a}}_{j}≔\lambda_{j}a_{j}$. It is easy to see that $\Lambda_{q}(\{{\tilde{a}}_{j}\})≲{\tilde{\Lambda }}_{q}(\{\lambda_{j}\})<\infty$.

Conversely, if $f=\sum_{j}{\tilde{a}}_{j}$ in *𝒮*′(ℝ^*n*^) with $\Lambda_{q}(\{{\tilde{a}}_{j}\})<\infty$, by defining

(97) $$\begin{array}{c} \lambda_{j}≔{||{\tilde{a}}_{j}||}_{L_{\varphi }^{q}(x_{j}+B_{\ell_{j}})}{||\chi_{x_{j}+B_{\ell_{j}}}||}_{L^{\varphi }({\mathbb{R}}^{n})},\\ a_{j}≔{\tilde{a}}_{j}{||{\tilde{a}}_{j}||}_{L_{\varphi }^{q}\left(x_{j}+B_{\ell_{j}}\right)}^{-1}{||\chi_{x_{j}+B_{\ell_{j}}}||}_{L^{\varphi }({\mathbb{R}}^{n})}^{-1}, \end{array}$$

we see that *f* = ∑_*j*_ *λ*_*j*_ *a*_*j*_ and ${\tilde{\Lambda }}_{q}(\{\lambda_{j}\})=\Lambda_{q}(\{{\tilde{a}}_{j}\})<\infty$. Thus, the above claim holds true.

(ii) If *φ* is as in (15) with an anisotropic *A*_*∞*_(ℝ^*n*^) Muckenhoupt weight *w* and Φ(*t*)≔*t* ^*p*^ for all *t* ∈ [0, *∞*) with *p* ∈ (0, 1], then the atomic space *H*_*A*_^*φ*,*q*,*s*^(ℝ^*n*^) is just the weighted anisotropic atomic Hardy space introduced in [6].

The following lemma shows that anisotropic (*φ*, *q*, *s*)-atoms of Musielak-Orlicz type are in *H*_*A*_^*φ*^(ℝ^*n*^).

**Lemma 32**

Let (*φ*, *q*, *s*) be an anisotropic admissible triplet and let *m* ∈ [*s*, *∞*)∩*ℤ*_+_. Then there exists a positive constant *C*≔*C*(*φ*, *q*, *s*, *m*) such that, for any anisotropic (*φ*, *q*, *s*)-atom *a* associated with some *x*_0_ + *B*_*j*_,

(98) $$\begin{array}{c} \int_{{\mathbb{R}}^{n}}\varphi \left(x,a_{m}^{*}\left(x\right)\right)dx\leq C\varphi \left(x_{0}+B_{j},{||a||}_{L_{\varphi }^{q}(x_{0}+B_{j})}\right), \end{array}$$

and hence ||*a*||_*H*_*m*,*A*_^*φ*^(ℝ^*n*^)_ ≤ *C*.

**Proof**

The case *q* = *∞* is easy. We just consider *q* ∈ (*q*(*φ*), *∞*). Now let us write

(99) ∫ℝnφ(x,am∗(x))dx=∫x0+Bj+σφ(x,am∗(x))dx+∫(x0+Bj+σ)∁⋯=:I+II.

By using Lemma 10, the proof of I≲*φ*(*x*_0_ + *B*_*j*_, ||*a*||_*L*_*φ*_^*q*^(*x*_0_+*B*_*j*_)_) is similar to that of [20, Lemma 5.1], the details being omitted.

To estimate II, we claim that, for all *ℓ* ∈ *ℤ*_+_ and *x* ∈ *x*_0_ + (*B*_*j*+*σ*+*ℓ*+1_∖*B*_*j*+*σ*+*ℓ*_),

(100) $$\begin{array}{c} a_{m}^{*}\left(x\right)≲{||a||}_{L_{\varphi }^{q}(x_{0}+B_{j})}{\left[b{\left(\lambda_{-}\right)}^{s+1}\right]}^{-\ell }, \end{array}$$

where *s* ≥ ⌊(*q*(*φ*)/*i*(*φ*) − 1)ln⁡*b*/ln⁡(*λ*_−_)⌋. If this claim is true, choosing $\tilde{q}>q(\varphi )$ and *p* < *i*(*φ*) such that $b^{-\tilde{q}+p}{\left(\lambda_{-}\right)}^{(s+1)p}>1$, then, by $\varphi \in {𝔸}_{\tilde{q}}(A)$ and Lemma 10, we have

(101) II≲∑ℓ=0∞∫x0+(Bj+ℓ+σ+1∖Bj+ℓ+σ)[b(λ−)s+1]−ℓp   ×φ(x,||a||Lφq(x0+Bj))dx≲φ(x0+Bj,||a||Lφq(x0+Bj)) ×∑ℓ=0∞[b−q~+p(λ−)(s+1)p]−ℓ≲φ(x0+Bj,||a||Lφq(x0+Bj)).

Combining the estimates for I and II, we obtain (98).

To prove the estimate (100), we borrow some techniques from the proof of Theorem 4.2 in [9]. By Hölder's inequality, *φ* ∈ *𝔸*_*q*_(*A*), and

(102) $$\begin{array}{c} {\left\{\int_{x_{0}+B_{j}}{\left[\varphi \left(y,\lambda \right)\right]}^{-q^{\prime }/q}dy\right\}}^{1/q^{\prime }}\leq \frac{b^{j}}{{\left[\varphi \left(x_{0}+B_{j},\lambda \right)\right]}^{1/q}}, \end{array}$$

we obtain

(103) ∫x0+Bj|a(y)|dy≤{∫x0+Bj|a(y)|qφ(y,λ)dy}1/q ×(∫x0+Bj[φ(y,λ)]−q′/qdx)1/q′≲bj||a||Lφq(x0+Bj).

Let *x* ∈ *x*_0_ + (*B*_*j*+*ℓ*+*σ*+1_∖*B*_*j*+*ℓ*+*σ*_), *k* ∈ *ℤ*, and *ϕ* ∈ *𝒮*_*s*_(ℝ^*n*^). For *j* + *k* > 0 and *y* ∈ *x*_0_ + *B*_*j*_, we have *ρ*(*A* ^*k*^(*x* − *y*)) ≳ *b* ^*j*+*k*+*ℓ*^. Observe that *b*(*λ*_−_)^*s*+1^ ≤ *b* ^*s*+2^. By this, (103), *ϕ* ∈ *𝒮*_*s*_(ℝ^*n*^), and *j* + *k* > 0, we conclude that

(104) |a∗ϕk(x)|≤bk∫x0+Bj|a(y)||ϕ(Ak(x−y))|dy≲b−(s+2)(j+k+ℓ)bj+k||a||Lφq(x0+Bj)≲[b(λ−)s+1]−ℓ||a||Lφq(x0+Bj).

For *j* + *k* ≤ 0, let *P* be the Taylor expansion of *ϕ* at the point *A* ^−*k*^(*x* − *x*_0_) of order *s*. Thus, by the Taylor remainder theorem and |*A* ^(*j*+*k*)^ *z* | ≲(*λ*_−_)^(*j*+*k*)^ | *z*| for all *z* ∈ ℝ^*n*^ (see [9, Section 2]), we see that

(105) sup⁡y∈x0+Bj|ϕ(Ak(x−y))−P(Ak(x−y))| ≲sup⁡z∈Bj+k sup⁡|α|=s+1|∂αϕ(Ak(x−x0)+z)||z|s+1 ≲(λ−)(s+1)(j+k)sup⁡z∈Bj+k[1+ρ(Ak(x−x0)+z)]−(s+2) ≲(λ−)(s+1)(j+k)min⁡{1,b−(s+2)(j+k+ℓ)},

where, in the last step, we used (8) and the fact that

(106) $$\begin{array}{c} A^{k}\left(x-x_{0}\right)+B_{j+k}\subset {\left(B_{j+k+\ell +\sigma }\right)}^{∁}+B_{j+k}\subset {\left(B_{j+k+\ell }\right)}^{∁}, \end{array}$$

since *ℓ* ≥ 0. By this, (103), *j* + *k* ≤ 0, and the fact that *a* has vanishing moments up to order *s*, we find that

(107) |a∗ϕk(x)| ≤bk∫x0+Bj|a(y)||ϕ(Ak(x−y))−P(Ak(x−y))|dy ≲||a||Lφq(x0+Bj)(λ−)(s+1)(j+k)bj+kmin⁡{1,b−(s+2)(j+k+ℓ)}.

Observe that, when *j* + *k* + *ℓ* > 0, by *b*(*λ*_−_)^*s*+1^ ≤ *b* ^*s*+2^, we know that

(108) $$\begin{array}{c} |a*\phi_{k}\left(x\right)|≲{\left[{\left(\lambda_{-}\right)}^{\left(s+1\right)}b\right]}^{-\ell }{||a||}_{L_{\varphi }^{q}(x_{0}+B_{j})}. \end{array}$$

Finally, when *j* + *k* + *ℓ* ≤ 0, from (107), we immediately deduce (108). This shows that (108) holds for all *j* + *k* ≤ 0. Combining this with (104), and taking supremum over *k* ∈ *ℤ*, we see that

(109) $$\begin{array}{c} \underset{\phi \in {𝒮}_{s}({\mathbb{R}}^{n})\;}{\sup}\underset{k\in \mathbb{Z}}{\sup}|\phi_{k}*a\left(x\right)|≲{\left[{\left(\lambda_{-}\right)}^{\left(s+1\right)}b\right]}^{-\ell }{||a||}_{L_{\varphi }^{q}(x_{0}+B_{j})}. \end{array}$$

From this estimate and *a*_m_*(*x*)≲sup⁡_*ϕ*∈*𝒮*_*s*_(ℝ^*n*^)_sup⁡_*k*∈*ℤ*_ | *a*∗*ϕ*_*k*_(*x*)| (see [9, Propostion 3.10]), we further deduce (100) and hence complete the proof of Lemma 37.

Then, by using Lemma 32, together with an argument similar to that used in the proof of [20, Theorem 5.1], we obtain the following theorem, the details being omitted.

**Theorem 33**

Let (*φ*, *q*, *s*) be an admissible triplet and let *m* ∈ [*s*, *∞*)∩*ℤ*_+_. Then

(110) $$\begin{array}{c} H_{A}^{\varphi ,q,s}\left({\mathbb{R}}^{n}\right)\subset H_{m,A}^{\varphi }\left({\mathbb{R}}^{n}\right) \end{array}$$

and the inclusion is continuous.

To obtain the conclusion *H*_*m*,*A*_^*φ*^(ℝ^*n*^) ⊂ *H*_*A*_^*φ*,*q*,*s*^(ℝ^*n*^), we use the Calderón-Zygmund decomposition obtained in Section 4. Let *φ* be an anisotropic growth function, let *m* ≥ ⌊*q*(*φ*)ln⁡*b*/[*i*(*φ*)ln⁡*λ*]⌋, and let *f* ∈ *H*_*m*,*A*_^*φ*^(ℝ^*n*^). For each *k* ∈ *ℤ*, as in Definition 19, *f* has a Calderón-Zygmund decomposition of degree *s* and height *λ* = 2^*k*^ associated with *f*_m_* as follows:

(111) $$\begin{array}{c} f=g^{k}+\underset{i}{\sum }b_{i}^{k}, \end{array}$$

where

(112) $$\begin{array}{c} \Omega_{k}≔\left\{x:f_{m}^{*}\left(x\right)>2^{k}\right\},\;\;b_{i}^{k}≔\left(f-P_{i}^{k}\right)\zeta_{i}^{k},\\ B_{i}^{k}≔x_{i}^{k}+B_{\ell_{i}^{k}}. \end{array}$$

Recall that, for fixed *k* ∈ *ℤ*, {*x*_*i*_^*k*^}_*i*_≔{*x*_*i*_}_*i*_ is a sequence in *Ω*_*k*_ and {*ℓ*_*i*_^*k*^}_*i*_≔{*ℓ*_*i*_}_*i*_ is a sequence of integers such that (65) through (69) hold for *Ω*≔*Ω*_*k*_, {*ζ*_*i*_^*k*^}_*i*_≔{*ζ*_*i*_}_*i*_ are given by (70), and {*P*_*i*_^*k*^}_*i*_≔{*P*_*i*_}_*i*_ are projections of *f* onto *𝒫*_*s*_(ℝ^*n*^) with respect to the norms given by (71). Moreover, for each *k* ∈ *ℤ* and *i*, *j*, let *P*_*i*,*j*_^*k*+1^ be the orthogonal projection of (*f* − *P*_*j*_^*k*+1^)*ζ*_*i*_^*k*^ onto *𝒫*_*s*_(ℝ^*n*^) with respect to the norm associated with *ζ*_*j*_^*k*+1^ given by (71), namely, the unique element of *𝒫*_*s*_(ℝ^*n*^) such that, for all *Q* ∈ *𝒫*_*s*_(ℝ^*n*^),

(113) ∫ℝn[f(x)−Pjk+1(x)]ζik(x)Q(x)ζjk+1(x)dx  =∫ℝnPi,jk+1(x)Q(x)ζjk+1(x)dx.

For convenience, let ${\hat{B}}_{i}^{k}≔x_{i}^{k}+B_{\ell_{i}^{k}+\sigma }$.

Lemmas 34 through 36 are just [9, Lemmas 5.1 through 5.3], respectively.

**Lemma 34**

The following hold true.

- If ${\hat{B}}_{j}^{k+1}\cap {\hat{B}}_{i}^{k}\neq \emptyset$, then *ℓ*_*j*_^*k*+1^ ≤ *ℓ*_*i*_^*k*^ + *σ* and ${\hat{B}}_{j}^{k+1}\subset x_{i}^{k}+B_{\ell_{i}^{k}+4\sigma }$.
- For any *i*, $\#\{j:{\hat{B}}_{j}^{k+1}\cap {\hat{B}}_{i}^{k}\neq \emptyset \}\leq 2L$, where *L* is as in (69).

**Lemma 35**

There exists a positive constant *C*_10_, independent of *f*, such that, for all *i*, *j*, and *k* ∈ *ℤ*,

(114) $$\begin{array}{c} \underset{y\in {\mathbb{R}}^{n}}{\sup}|P_{i,j}^{k+1}\left(y\right)\zeta_{j}^{k+1}\left(y\right)|\leq C_{10}\underset{y\in U}{\sup}f_{m}^{*}\left(y\right)\leq C_{10}2^{k+1}, \end{array}$$

where *U*≔(*x*_*j*_^*k*+1^ + *B*_*ℓ*_*j*_^*k*+1^+4*σ*+1_)∩(*Ω*_*k*+1_)^∁^.

**Lemma 36**

For every *k* ∈ *ℤ*, ∑_*i*_∑_*j*_ *P*_*i*,*j*_^*k*+1^ *ζ*_*j*_^*k*+1^ = 0, where the series converges pointwise and also in *𝒮*′(ℝ^*n*^).

The proof of the following lemma is similar to that of [20, Lemma 5.4], the details being omitted.

**Lemma 37**

Let *m* ∈ *ℕ* and let *f* ∈ *H*_*m*,*A*_^*φ*^(ℝ^*n*^). Then, for any *λ* ∈ (0, *∞*), there exists a positive constant *C*, independent of *f* and *λ*, such that

(115) $$\begin{array}{c} \underset{k\in \mathbb{Z}}{\sum }\varphi \left(\Omega_{k},\frac{2^{k}}{\lambda }\right)\leq C\int_{{\mathbb{R}}^{n}}\varphi \left(x,\frac{f_{m}^{*}\left(x\right)}{\lambda }\right)dx. \end{array}$$

The following lemma establishes the atomic decompositions for a dense subspace of *H*_*m*,*A*_^*φ*^(ℝ^*n*^).

**Lemma 38**

Let *m* ≥ *s* ≥ ⌊*q*(*φ*)ln⁡*b*/[*i*(*φ*)ln⁡*λ*]⌋ and let *q* ∈ (*q*(*φ*), *∞*). Then, for any *f* ∈ *L*_*φ*(·,1)_^*q*^(ℝ^*n*^)∩*H*_*m*,*A*_^*φ*^(ℝ^*n*^), there exists a sequence {*a*_*i*_^*k*^}_*k*∈*ℤ*,*i*_ of multiples of (*φ*, *∞*, *s*)-atoms such that *f* = ∑_*k*∈*ℤ*_∑_*i*_ *a*_*i*_^*k*^ converges almost everywhere and also in *𝒮*′(ℝ^*n*^), and

(116) $$\begin{array}{c} \mathrm{supp}a_{i}^{k}\subset x_{i}^{k}+B_{\ell_{i}^{k}+4\sigma }\;\forall k\in \mathbb{Z},i, \end{array}$$

(117) $$\begin{array}{c} \Omega_{k}=\cup_{i}\left(x_{i}^{k}+B_{\ell_{i}^{k}+4\sigma }\right)\;\forall k\in \mathbb{Z}, \end{array}$$

(118) (xik+Bℓik−2σ)∩(xjk+Bℓjk−2σ)=∅∀k∈ℤ,  i,j  with  i≠j.

Moreover, there exists a positive constant *C*, independent of *f*, such that, for all *k* ∈ *ℤ* and *i*,

(119) $$\begin{array}{c} |a_{i}^{k}|\leq C2^{k} \end{array}$$

and, for any *λ* ∈ (0, *∞*),

(120) ∑k∈ℤ∑iφ(xik+Bℓik+4σ,||aik||L∞(ℝn)λ) ≤C∫ℝnφ(x,fm∗(x)λ)dx.

**Proof**

Let *f* ∈ *H*_*m*,*A*_^*φ*^(ℝ^*n*^)∩*L*_*φ*(·,1)_^*q*^(ℝ^*n*^). For each *k* ∈ *ℤ*, *f* has a Calderón-Zygmund decomposition of degree *s* ≥ ⌊*q*(*φ*)ln⁡*b*/[*i*(*φ*)ln⁡(*λ*_−_)]⌋ and height 2^*k*^ associated with *f*_m_*, *f* = *g* ^*k*^ + ∑_*i*_ *b*_*i*_^*k*^ as above. The conclusions (117) and (118) follow immediately from (65) and (66). By (76) of Lemma 24 and Proposition 6, we know that *g* ^*k*^ → *f* in both *H*_*m*,*A*_^*φ*^(ℝ^*n*^) and *𝒮*′(ℝ^*n*^) as *k* → *∞*. It follows, from Lemma 27(ii), that ||*g* ^*k*^||_*L*^*∞*^(ℝ^*n*^)_ → 0 as *k* → −*∞*, which further implies that *g* ^*k*^ → 0 almost everywhere as *k* → −*∞*, and, moreover, by the fact that *L* ^*∞*^(ℝ^*n*^) is continuously embedding into *𝒮*′(ℝ^*n*^) (see [6, Lemma 2.8]), we conclude that *g* ^*k*^ → 0 in *𝒮*′(ℝ^*n*^) as *k* → −*∞*. Therefore, we obtain

(121) $$\begin{array}{c} f=\underset{k\in \mathbb{Z}}{\sum }\left(g^{k+1}-g^{k}\right) \end{array}$$

in *𝒮*′(ℝ^*n*^).

Since supp⁡(∑_*i*_ *b*_*i*_^*k*^) ⊂ *Ω*_*k*_ and *φ*(*Ω*_*k*_, 1) → 0 as *k* → *∞*, then *g* ^*k*^ → *f* almost everywhere as *k* → *∞*. Thus, (121) also holds almost everywhere. By Lemma 36 and ∑_*i*_ *ζ*_*i*_^*k*^ *b*_*j*_^*k*+1^ = *χ*_*Ω*_*k*__ *b*_*j*_^*k*+1^ = *b*_*j*_^*k*+1^ for all *j*, we see that

(122) gk+1−gk=(f−∑jbjk+1)−(f−∑jbjk)=∑jbjk−∑jbjk+1+∑i(∑jPi,jk+1ζjk+1)=∑i[bik−∑j(ζikbjk+1−Pi,jk+1ζjk+1)]=:∑iaik,

where all the series converge in *𝒮*′(ℝ^*n*^) and almost everywhere. Furthermore,

(123) $$\begin{array}{c} a_{i}^{k}=\left(f-P_{i}^{k}\right)\zeta_{i}^{k}-\underset{j}{\sum }\left[\left(f-P_{j}^{k+1}\right)\zeta_{i}^{k}-P_{i,j}^{k+1}\right]\zeta_{j}^{k+1}. \end{array}$$

By definitions of *P*_*i*_^*k*^ and *P*_*i*,*j*_^*k*+1^, for all *Q* ∈ *𝒫*_*s*_(ℝ^*n*^), we have

(124) $$\begin{array}{c} \int_{{\mathbb{R}}^{n}}a_{i}^{k}\left(x\right)Q\left(x\right)dx=0. \end{array}$$

Moreover, since ∑_*j*_ *ζ*_*j*_^*k*+1^ = *χ*_*Ω*_*k*+1__, we rewrite (123) into

(125) aik=fχ(Ωk+1)∁ζik−Pikζik +∑jPjk+1ζikζjk+1+∑jPi,jk+1ζjk+1.

By Lemma 17, we know that |*f*(*x*)| ≤ *f*_m_*(*x*) ≤ 2^*k*+1^ for almost every *x* ∈ (*Ω*_*k*+1_)^∁^, and, by Lemmas 21, 34(ii), and 35, we find that

(126) $$\begin{array}{c} {||a_{i}^{k}||}_{L^{\infty }({\mathbb{R}}^{n})}≲2^{k}. \end{array}$$

Recall that *P*_*i*,*j*_^*k*+1^ ≠ 0 implies ${\hat{B}}_{j}^{k+1}\cap {\hat{B}}_{i}^{k}\neq \emptyset$, and hence, by Lemma 34(i), we see that $\mathrm{supp}\zeta_{j}^{k+1}\subset {\hat{B}}_{j}^{k+1}\subset x_{i}^{k}+B_{\ell_{i}^{k}+4\sigma }$. Therefore, by applying (123), we further conclude that

(127) $$\begin{array}{c} \mathrm{supp}a_{i}^{k}\subset x_{i}^{k}+B_{\ell_{i}^{k}+4\sigma }. \end{array}$$

Obviously, (126) and (127) imply (119) and (116), respectively. Moreover, by (124), (126), and (127), we know that *a*_*i*_^*k*^ is a multiple of a (*φ*, *∞*, *s*)-atom. By Lemma 10, (118), (126), uniformly upper type 1 property of *φ*, and Lemma 37, for any *λ* ∈ (0, *∞*), we have

(128) ∑k∈ℤ∑iφ(xik+Bℓik+4σ,||aik||L∞(ℝn)λ)  ≲∑k∈ℤ∑iφ(xik+Bℓik−2σ,||aik||L∞(ℝn)λ)  ≲∑k∈ℤφ(Ωk,2kλ)≲∫ℝnφ(x,fm∗(x)λ)dx,

which gives (120). This finishes the proof of Lemma 38.

The following Lemma 39 is just [20, Lemma 4.3(ii)].

**Lemma 39**

Let *φ* be an anisotropic growth function. For any given positive constant *c*, there exists a positive constant *C* such that, for some *λ* ∈ (0, *∞*), the inequality ∑_*j*_ *φ*(*x*_*j*_ + *B*_*ℓ*_*j*__, *t*_*j*_/*λ*) ≤ *c* implies that

(129) $$\begin{array}{c} \inf\left\{\alpha \in \left(0,\infty \right):\underset{j}{\sum }\varphi \left(x_{j}+B_{\ell_{j}},\frac{t_{j}}{\alpha }\right)\leq 1\right\}\leq C\lambda . \end{array}$$

**Theorem 40**

Let *q*(*φ*) be as in (13). If *m* ≥ *s* ≥ ⌊*q*(*φ*)ln⁡*b*/[*i*(*φ*)ln⁡*λ*]⌋ and *q* ∈ (*q*(*φ*), *∞*], then *H*_*A*_^*φ*,*q*,*s*^(ℝ^*n*^) = *H*_*m*,*A*_^*φ*^(ℝ^*n*^) = *H*_*A*_^*φ*^(ℝ^*n*^) with equivalent (quasi)norms.

**Proof**

Observe that, by (103), Definition 30, and Theorem 33, it holds true that

(130) $$\begin{array}{c} H_{A}^{\varphi ,\infty ,s}\left({\mathbb{R}}^{n}\right)\subset H_{A}^{\varphi ,q,s}\left({\mathbb{R}}^{n}\right)\subset H_{A}^{\varphi }\left({\mathbb{R}}^{n}\right)\subset H_{m,A}^{\varphi }\left({\mathbb{R}}^{n}\right), \end{array}$$

where *m* ≥ *s* ≥ ⌊*q*(*φ*)ln⁡*b*/[*i*(*φ*)ln⁡*λ*]⌋, and all the inclusions are continuous. Thus, to finish the proof of Theorem 40, it suffices to prove that, for all *f* ∈ *H*_*m*,*A*_^*φ*^(ℝ^*n*^) with *m* ≥ *s* ≥ ⌊*q*(*φ*)ln⁡*b*/[*i*(*φ*)ln⁡*λ*]⌋, ||*f*||_*H*_*A*_^*φ*,*∞*,*s*^(ℝ^*n*^)_≲||*f*||_*H*_*m*,*A*_^*φ*^(ℝ^*n*^)_, which implies that *H*_*m*,*A*_^*φ*^(ℝ^*n*^) ⊂ *H*_*A*_^*φ*,*∞*,*s*^(ℝ^*n*^).

To this end, let *f* ∈ *H*_*m*,*A*_^*φ*^(ℝ^*n*^)∩*L*_*φ*(·,1)_^*q*^(ℝ^*n*^); by Lemma 38, we obtain

(131) ∑k∈ℤ∑iφ(xik+Bℓik+4σ,||aik||L∞(ℝn)||f||Hm,Aφ(ℝn))  ≲∫ℝnφ(x,fm∗(x)||f||Hm,Aφ(ℝn))dx.

Consequently, by Lemma 39, we see that

(132) $$\begin{array}{c} {||f||}_{H_{A}^{\varphi ,\infty ,s}({\mathbb{R}}^{n})}\leq \Lambda_{\infty }\left(\left\{a_{i}^{k}\right\}\right)≲{||f||}_{H_{m,A}^{\varphi }({\mathbb{R}}^{n})}. \end{array}$$

Let *f* ∈ *H*_*m*,*A*_^*φ*^(ℝ^*n*^). By Corollary 28, there exists a sequence {*f*_*k*_}_*k*∈*ℕ*_ of functions in *H*_*m*,*A*_^*φ*^(ℝ^*n*^)∩*L*_*φ*(·,1)_^*q*^(ℝ^*n*^) such that ||*f*_*k*_||_*H*_*m*,*A*_^*φ*^(ℝ^*n*^)_ ≤ 2^−*k*^||*f*||_*H*_*m*,*A*_^*φ*^(ℝ^*n*^)_ and *f* = ∑_*k*∈*ℕ*_ *f*_*k*_ in *H*_*m*,*A*_^*φ*^(ℝ^*n*^). By Lemma 38, for each *k* ∈ *ℕ*, *f*_*k*_ has an atomic decomposition *f*_*k*_ = ∑_*i*∈*ℕ*_ *d*_*i*_^*k*^ in *𝒮*′(ℝ^*n*^), where {*d*_*i*_^*k*^}_*i*∈*ℕ*_ are multiples of (*φ*, *∞*, *s*)-atoms with supp⁡*d*_*i*_^*k*^ ⊂ *x*_*i*_^*k*^ + *B*_*ℓ*_*i*_^*k*^_. Since

(133) ∑k∈ℕ ∑i∈ℕφ(xik+Bℓik,||dik||L∞(ℝn)||f||Hm,Aφ(ℝn))  ≤∑k∈ℕ ∑i∈ℕφ(xik+Bℓik,||dik||L∞(ℝn)2k||fk||Hm,Aφ(ℝn))  ≲∑k∈ℕ1(2k)p≲1,

then, by Lemma 39, we further see that *f* = ∑_*k*∈*ℕ*_∑_*i*∈*ℕ*_ *d*_*i*_^*k*^ ∈ *H*_*A*_^*φ*,*∞*,*s*^(ℝ^*n*^) and

(134) $$\begin{array}{c} {||f||}_{H_{A}^{\varphi ,\infty ,s}({\mathbb{R}}^{n})}\leq \Lambda_{\infty }\left(\left\{a_{i}^{k}\right\}\right)≲{||f||}_{H_{m,A}^{\varphi }({\mathbb{R}}^{n})}, \end{array}$$

which completes the proof of Theorem 40.

For simplicity, from now on, we denote simply by *H*_*A*_^*φ*^(ℝ^*n*^) the anisotropic Hardy space *H*_*m*,*A*_^*φ*^(ℝ^*n*^) of Musielak-Orlicz type with *m* ≥ *m*(*φ*).

## 6. Finite Atomic Decompositions and Their Applications

The goal of this section is to obtain the finite atomic decomposition characterization of *H*_*A*_^*φ*^(ℝ^*n*^), and, as an application, a bounded criterion on *H*_*A*_^*φ*^(ℝ^*n*^) of quasi-Banach space-valued sublinear operators is also obtained.

### 6.1. Finite Atomic Decompositions

In this subsection, we prove that, for any given finite linear combination of atoms when *q* < *∞* (or continuous atoms when *q* = *∞*), its norm in *H*_*A*_^*φ*^(ℝ^*n*^) can be achieved via all its finite atomic decompositions. This extends the conclusion [39, Theorem 3.1] by Meda et al. to the setting of anisotropic Hardy spaces of Musielak-Orlicz type.

**Definition 41**

Let (*φ*, *q*, *s*) be an admissible triplet. Denote by *H*_*A*,fin_^*φ*,*q*,*s*^(ℝ^*n*^) the* set of all finite linear combinations of multiples of *(*φ*, *q*, *s*)*-atoms*, and the* norm* of *f* in *H*_*A*,fin_^*φ*,*q*,*s*^(ℝ^*n*^) is defined by

(135) ||f||HA,finφ,q,s(ℝn)≔inf⁡{Λq({aj}j=1k):f=∑j=1kaj,         k∈ℕ,{ai}i=1k are (φ,q,s)-atoms}.

Obviously, for any admissible triplet (*φ*, *q*, *s*), the set *H*_*A*,fin_^*φ*,*q*,*s*^(ℝ^*n*^) is dense in *H*_*A*_^*φ*,*q*,*s*^(ℝ^*n*^) with respect to the quasinorm ||·||_*H*_*A*_^*φ*,*q*,*s*^(ℝ^*n*^)_.

In order to obtain the finite atomic decomposition, we need the notion of the uniformly locally dominated convergence condition from [20]. An anisotropic growth function *φ* is said to satisfy the* uniformly locally dominated convergence condition* if the following holds: for any compact set *K* in ℝ^*n*^ and any sequence {*f*_*m*_}_*m*∈*ℕ*_ of measurable functions such that *f*_*m*_(*x*) tends to *f*(*x*) for almost every *x* ∈ ℝ^*n*^, if there exists a nonnegative measurable function *g* such that |*f*_*m*_(*x*)|≤*g*(*x*) for almost every *x* ∈ ℝ^*n*^ and

(136) $$\begin{array}{c} \underset{t\in \left(0,\infty \right)}{\sup}\int_{K}g\left(x\right)\frac{\varphi \left(x,t\right)}{\int_{K}\varphi \left(y,t\right)dy}dx<\infty , \end{array}$$

then

(137) sup⁡t∈(0,∞)∫K|fm(x)−f(x)|φ(x,t)∫Kφ(y,t)dydx⟶0as  m⟶∞.

We remark that the anisotropic growth functions *φ*(*x*, *t*)≔*t* ^*p*^/[log⁡(*e* + |*x*|) + log⁡(*e* + *t* ^*p*^)]^*p*^ for all *x* ∈ ℝ^*n*^ and *t* ∈ (0, *∞*) with *p* ∈ (0, 1) and *φ* as in (15) satisfy the* uniformly locally dominated convergence condition*; see [20, page 12].

**Theorem 42**

Let *φ* be an anisotropic growth function satisfying the uniformly locally dominated convergence condition, *q*(*φ*) as in (13), and (*φ*, *q*, *s*) an admissible triplet.

- If *q* ∈ (*q*(*φ*), *∞*), then ||·||_*H*_*A*,fin_^*φ*,*q*,*s*^(ℝ^*n*^)_ and ||·||_*H*_*A*_^*φ*^(ℝ^*n*^)_ are equivalent quasinorms on *H*_*A*,fin_^*φ*,*q*,*s*^(ℝ^*n*^).
- ||·||_*H*_*A*,fin_^*φ*,*∞*,*s*^(ℝ^*n*^)_ and ||·||_*H*_*A*_^*φ*^(ℝ^*n*^)_ are equivalent quasinorms on *H*_*A*,fin_^*φ*,*∞*,*s*^(ℝ^*n*^)∩*ℂ*(ℝ^*n*^).

**Proof**

Obviously, by Theorem 40, *H*_*A*,fin_^*φ*,*q*,*s*^(ℝ^*n*^) ⊂ *H*_*A*_^*φ*^(ℝ^*n*^) and, for all *f* ∈ *H*_*A*,fin_^*φ*,*q*,*s*^(ℝ^*n*^),

(138) $$\begin{array}{c} {||f||}_{H_{A}^{\varphi }({\mathbb{R}}^{n})}\leq {||f||}_{H_{A,\text{fin}}^{\varphi ,q,s}({\mathbb{R}}^{n})}. \end{array}$$

Thus, we only need to prove that, for all *f* ∈ *H*_*A*,fin_^*φ*,*q*,*s*^(ℝ^*n*^) when *q* ∈ (*q*(*φ*), *∞*) and for all *f* ∈ *H*_*A*,fin_^*φ*,*q*,*s*^(ℝ^*n*^)∩*ℂ*(ℝ^*n*^) when *q* = *∞*, ||*f*||_*H*_*A*,fin_^*φ*,*q*,*s*^(ℝ^*n*^)_≲||*f*||_*H*_*A*_^*φ*^(ℝ^*n*^)_.

Now we prove this by three steps.

*Step  1 (a new decomposition of f* ∈ *H*_*A*,*fin*_^*φ*,*q*,*s*^(ℝ^*n*^)). Assume that *q* ∈ (*q*(*φ*), *∞*]. Without loss of generality, we may assume that *f* ∈ *H*_*A*,fin_^*φ*,*q*,*s*^(ℝ^*n*^) and ||*f*||_*H*_*A*_^*φ*^(ℝ^*n*^)_ = 1. Notice that *f* has compact support. Suppose that supp⁡*f* ⊂ *B*≔*B*_*k*_0__ for some *k*_0_ ∈ *ℤ*, where *B*_*k*_0__ is as in Section 2. For each *k* ∈ *ℤ*, let

(139) $$\begin{array}{c} \Omega_{k}≔\left\{x\in {\mathbb{R}}^{n}:f^{*}\left(x\right)>2^{k}\right\}. \end{array}$$

We use the same notation as in Lemma 38. Since $f\in H_{A}^{\varphi }({\mathbb{R}}^{n})\cap L_{\varphi (\cdot ,1)}^{\tilde{q}}({\mathbb{R}}^{n})$, where $\tilde{q}≔q$ if *q* ∈ (*q*(*φ*), *∞*) and $\tilde{q}≔q(\varphi )+1$ if *q* = *∞*, by Lemma 38, there exists a sequence {*a*_*i*_^*k*^}_*k*∈*ℤ*,*i*_ of multiples of (*φ*, *∞*, *s*)-atoms such that *f* = ∑_*k*∈*ℤ*_∑_*i*_ *a*_*i*_^*k*^ holds almost everywhere and in *𝒮*′(ℝ^*n*^). Moreover, by *H*_*A*_^*φ*,*∞*,*s*^(ℝ^*n*^) ⊂ *H*_*A*_^*φ*,*q*,*s*^(ℝ^*n*^) and Theorem 40, we know that

(140) $$\begin{array}{c} \Lambda_{q}\left(\left\{a_{i}^{k}\right\}\right)\leq \Lambda_{\infty }\left(\left\{a_{i}^{k}\right\}\right)≲{||f||}_{H_{A}^{\varphi }({\mathbb{R}}^{n})}≲1. \end{array}$$

On the other hand, by Step  2 of the proof of [6, Theorem 6.2], we know that there exists a positive constant $\tilde{C}$, depending only on *m*(*φ*), such that $f^{*}(x)\leq \tilde{C}\;\inf_{y\in B}f^{*}(y)$ for all *x* ∈ (B*)^∁^≔(*B*_*k*_0_+4*σ*_)^∁^. Hence, for all *x* ∈ (B*)^∁^, we have

(141) f∗(x)≤C~ inf⁡y∈Bf∗(y)≤C~||χB||Lφ(ℝn)−1||f∗||Lφ(ℝn)≤C~||χB||Lφ(ℝn)−1.

We now denote by *k*′ the largest integer *k* such that $2^{k}<\tilde{C}{||\chi_{B}||}_{L^{\varphi }({\mathbb{R}}^{n})}^{-1}$. Then

(142) $$\begin{array}{c} \Omega_{k}\subset B^{*}:=B_{k_{0}+4\sigma }\;\forall k>k^{\prime }. \end{array}$$

Let *h*≔∑_*k*≤*k*′_∑_*i*_ *λ*_*i*_^*k*^ *a*_*i*_^*k*^ and let *ℓ*≔∑_*k*>*k*′_∑_*i*_ *λ*_*i*_^*k*^ *a*_*i*_^*k*^, where the series converge almost everywhere and in *𝒮*′(ℝ^*n*^). Clearly, *f* = *h* + *ℓ* and supp⁡*ℓ* ⊂ ⋃_*k*>*k*′_ *Ω*_*k*_ ⊂ B*, which, together with supp⁡*f* ⊂ B*, further yields supp⁡*h* ⊂ B*.

*Step  2 (prove h* *to be a multiple of a *(*φ*, *q*, *s*)*-atom)*. Notice that, for any *q* ∈ (*q*(*φ*), *∞*] and *q*_1_ ∈ (1, *q*/*q*(*φ*)), by Hölder's inequality and *φ* ∈ *𝔸*_*q*/*q*_1__(*A*), we have

(143) [1|B|∫B|f(x)|q1dx]1/q1  ≲[1φ(B,1)∫B|f(x)|qφ(x,1)dx]1/q  <∞.

Observing that supp⁡*f* ⊂ *B* and *f* has vanishing moments up to order *s*, we know that *f* is a multiple of a (1, *q*_1_, 0)-atom and therefore f* ∈ *L* ^1^(ℝ^*n*^). Then, by (142), (116), (117), and (119) of Lemmas 38 and 34(ii), for any |*α* | ≤*s*, we conclude that

(144) ∫ℝn∑k>k′∑i|λikaik(x)xα|dx≲∑k∈ℤ2k|Ωk|≲||f∗||L1(ℝn)<∞.

(Notice that *a*_*i*_^*k*^ in (119) is replaced by *λ*_*i*_^*k*^ *a*_*i*_^*k*^ here.) This, together with the vanishing moments of *a*_*i*_^*k*^, implies that *ℓ* has vanishing moments up to order *s* and, hence, so does *h* by *h* = *f* − *ℓ*. Using Lemma 34(ii), (119) of Lemma 38, and the facts that $2^{k^{\prime }}\leq \tilde{C}{||\chi_{B}||}_{L^{\varphi }({\mathbb{R}}^{n})}^{-1}$ and ||*χ*_*B*_||_*L*^*φ*^(ℝ^*n*^)_^−1^ ~ ||*χ*_B*_||_*L*^*φ*^(ℝ^*n*^)_^−1^, we obtain

(145) $$\begin{array}{c} {||h||}_{L^{\infty }({\mathbb{R}}^{n})}≲\underset{k\leq k^{\prime }}{\sum }2^{k}≲\tilde{C}{||\chi_{B}||}_{L^{\varphi }\left({\mathbb{R}}^{n}\right)}^{-1}≲{||\chi_{B^{*}}||}_{L^{\varphi }\left({\mathbb{R}}^{n}\right)}^{-1}. \end{array}$$

Thus, there exists a positive constant *C*_0_, independent of *f*, such that *h*/*C*_0_ is a (*φ*, *∞*, *s*)-atom, and, by Definition 30, it is also a (*φ*, *q*, *s*)-atom for any admissible triplet (*φ*, *q*, *s*).

*Step  3 (prove (i))*. Let *q* ∈ (*q*(*φ*), *∞*). We first show ∑_*k*>*k*′_∑_*i*_ *λ*_*i*_^*k*^ *a*_*i*_^*k*^ ∈ *L*_*φ*(·,1)_^*q*^(ℝ^*n*^). For any *x* ∈ ℝ^*n*^, since ℝ^*n*^ = ∪_*k*∈*ℤ*_(*Ω*_*k*_∖*Ω*_*k*+1_), there exists *j* ∈ *ℤ* such that *x* ∈ (*Ω*_*j*_∖*Ω*_*j*+1_). Since supp⁡*a*_*i*_^*k*^ ⊂ *B*_*ℓ*_*i*_^*k*^+*σ*_ ⊂ *Ω*_*k*_ ⊂ *Ω*_*j*+1_ for *k* > *j*, applying Lemma 34(ii) and (119) of Lemma 38, we conclude that, for all *x* ∈ (*Ω*_*j*_∖*Ω*_*j*+1_),

(146) $$\begin{array}{c} \underset{k>k^{\prime }}{\sum }\underset{i}{\sum }|\lambda_{i}^{k}a_{i}^{k}\left(x\right)|≲\underset{k\leq j}{\sum }2^{k}≲2^{j}≲f^{*}\left(x\right). \end{array}$$

By *f* ∈ *L*_*φ*_^*q*^(*B*) ⊂ *L*_*φ*_^*q*^(B*), we further have f* ∈ *L*_*φ*_^*q*^(B*). Since *φ* satisfies the uniformly locally dominated convergence condition, it follows that ∑_*k*>*k*′_∑_*i*_ *λ*_*i*_^*k*^ *a*_*i*_^*k*^ converges to *ℓ* in *L*_*φ*_^*q*^(B*).

Now, for any positive integer *K*, let

(147) $$\begin{array}{c} F_{K}≔\left\{\left(i,k\right):k>k^{\prime },\left|i\right|+\left|k\right|\leq K\right\} \end{array}$$

and let *ℓ*_*K*_≔∑_(*i*,*k*)∈*F*_*K*__ *λ*_*i*_^*k*^ *a*_*i*_^*k*^. Since ∑_*k*>*k*′_∑_*i*_ *λ*_*i*_^*k*^ *a*_*i*_^*k*^ converges in *L*_*φ*_^*q*^(B*), for any *ϵ* ∈ (0, 1), if *K* is large enough, we have that (*ℓ* − *ℓ*_*K*_)/*ϵ* is a (*φ*, *q*, *s*)-atom. Thus, *f* = *h* + *ℓ*_*K*_ + (*ℓ* − *ℓ*_*K*_) is a finite linear combination of atoms. By (120) of Lemma 38 and Step  2, we further find that

(148) $$\begin{array}{c} {||f||}_{H_{A,\text{fin}}^{\varphi ,q,s}({\mathbb{R}}^{n})}≲C_{0}+\Lambda_{q}\left({\left\{a_{i}^{k}\right\}}_{(i,k)\in F_{K}}\right)+\epsilon ≲1, \end{array}$$

which completes the proof of (i).

To prove (ii), assume that *f* is a continuous function in *H*_*A*,fin_^*φ*,*∞*,*s*^(ℝ^*n*^); then *a*_*i*_^*k*^ is also continuous by examining its definition (see (123)). Since f*(*x*) ≤ *C*_*n*,*m*(*φ*)_||*f*||_*L*^*∞*^(ℝ^*n*^)_ for any *x* ∈ ℝ^*n*^, where the positive constant *C*_*n*,*m*(*φ*)_ only depends on *n* and *m*(*φ*), it follows that the level set *Ω*_*k*_ is empty for all *k* satisfying that 2^*k*^ ≥ *C*_*n*,*m*(*φ*)_||*f*||_*L*^*∞*^(ℝ^*n*^)_. We denote by *k*′′ the largest integer for which the above inequality does not hold. Then the index *k* in the sum defining *ℓ* will run only over *k*′ < *k* ≤ *k*′′.

Let *ϵ* ∈ (0, *∞*). Since *f* is uniformly continuous, it follows that there exists a *δ* ∈ (0, *∞*) such that if *ρ*(*x* − *y*) < *δ*, then |*f*(*x*) − *f*(*y*)|<*ϵ*. Write *ℓ* = *ℓ*_1_^*ϵ*^ + *ℓ*_2_^*ϵ*^ with *ℓ*_1_^*ϵ*^≔∑_(*i*,*k*)∈*F*_1__ *λ*_*i*_^*k*^ *a*_*i*_^*k*^ and *ℓ*_2_^*ϵ*^≔∑_(*i*,*k*)∈*F*_2__ *λ*_*i*_^*k*^ *a*_*i*_^*k*^, where

(149) $$\begin{array}{c} F_{1}≔\left\{\left(i,k\right):b^{\ell_{i}^{k}+\sigma }\geq \delta ,k^{\prime }<k\leq k^{\prime \prime }\right\} \end{array}$$

and *F*_2_≔{(*i*, *k*) : *b* ^*ℓ*_*i*_^*k*^+*σ*^ < *δ*, *k*′ < *k* ≤ *k*′′}.

On the other hand, for any fixed integer *k* ∈ (*k*′, *k*′′], by (118) of Lemma 38 and *Ω*_*k*_ ⊂ B*, we see that *F*_1_ is a finite set and hence *ℓ*_1_^*ϵ*^ is continuous. Furthermore, from Step  5 of the proof of [6, Theorem 6.2], it follows that |*ℓ*_2_^*ϵ*^ | ≤*ϵ*(*k*′′ − *k*′). Since *ϵ* is arbitrary, we can hence split *ℓ* into a continuous part and a part that is uniformly arbitrarily small. This fact implies that *ℓ* is continuous. Thus, *h* = *f* − *ℓ* is a multiple of a continuous (*φ*, *∞*, *s*)-atom by Step  2.

Now we can give a finite atomic decomposition of *f*. Let us use again the splitting *ℓ*≔*ℓ*_1_^*ϵ*^ + *ℓ*_2_^*ϵ*^. By (140), the part *ℓ*_1_^*ϵ*^ is a finite sum of multiples of (*φ*, *∞*, *s*)-atoms and

(150) $$\begin{array}{c} {||\ell_{1}^{\epsilon }||}_{H_{A,\text{fin}}^{\varphi ,\infty ,s}({\mathbb{R}}^{n})}\leq \Lambda_{\infty }\left(\left\{a_{i}^{k}\right\}\right)≲{||f||}_{H_{A}^{\varphi }({\mathbb{R}}^{n})}\sim 1. \end{array}$$

Notice that *ℓ*, *ℓ*_1_^*ϵ*^ are continuous and have vanishing moments up to order *s* and, hence, so does *ℓ*_2_^*ϵ*^ = *ℓ* − *ℓ*_1_^*ϵ*^. Moreover, supp⁡*ℓ*_2_^*ϵ*^ ⊂ B* and ||*ℓ*_2_^*ϵ*^||_*L*^*∞*^(ℝ^*n*^)_ ≤ *C*_1_(*k*′′ − *k*′)*ϵ*. Thus, we can choose *ϵ* small enough such that *ℓ*_2_^*ϵ*^ becomes a sufficient small multiple of a continuous (*φ*, *∞*, *s*)-atom; that is,

(151) $$\begin{array}{c} \ell_{2}^{\epsilon }=\lambda^{\epsilon }a^{\epsilon }\;\text{with}\;\;\lambda^{\epsilon }≔C_{1}\left(k^{\prime \prime }-k^{\prime }\right)\epsilon {||\chi_{B^{*}}||}_{L^{\varphi }({\mathbb{R}}^{n})},\\ a^{\epsilon }≔\frac{{||\chi_{B^{*}}||}_{L^{\varphi }({\mathbb{R}}^{n})}^{-1}}{C_{1}\left(k^{\prime \prime }-k^{\prime }\right)\epsilon }. \end{array}$$

Therefore, *f* = *h* + *ℓ*_1_^*ϵ*^ + *ℓ*_2_^*ϵ*^ is a finite linear combination of continuous atoms. Then, by (150) and the fact that *h*/*C*_0_ is a (*φ*, *∞*, *s*)-atom, we have

(152) ||f||HA,finφ,∞,s(ℝn)≲||h||HA,finφ,∞,s(ℝn) +||ℓ1ϵ||HA,finφ,∞,s(ℝn)+||ℓ2ϵ||HA,finφ,∞,s(ℝn)≲1.

This finishes the proof of (ii) and hence Theorem 42.

### 6.2. Applications

As an application of the finite atomic decompositions obtained in Theorem 42, we establish the boundedness on *H*_*A*_^*φ*^(ℝ^*n*^) of quasi-Banach-valued sublinear operators.

Recall that a* quasi-Banach space* *ℬ* is a vector space endowed with a quasinorm ||·||_*ℬ*_ which is nonnegative, nondegenerate (i.e., ||*f*||_*ℬ*_ = 0 if and only if *f* = 0), and homogeneous and obeys the quasitriangle inequality; that is, there exists a constant *K* ∈ [1, *∞*) such that, for all *f*, *g* ∈ *ℬ*, ||*f*+*g*||_*ℬ*_ ≤ *K*(||*f*||_*ℬ*_ + ||*g*||_*ℬ*_).

**Definition 43**

Let *γ* ∈ (0, 1]. A quasi-Banach space *ℬ*_*γ*_ with the quasinorm ||·||_*ℬ*_*γ*__ is a *γ-quasi-Banach space* if ||*f*+*g*||_*ℬ*_*γ*__^*γ*^ ≤ ||*f*||_*ℬ*_*γ*__^*γ*^ + ||*g*||_*ℬ*_*γ*__^*γ*^ for all *f*, *g* ∈ *ℬ*_*γ*_.

Notice that any Banach space is a 1-quasi-Banach space, and the quasi-Banach spaces *ℓ* ^*p*^, *L*_*w*_^*p*^(ℝ^*n*^), and *H*_*w*_^*p*^(ℝ^*n*^; *A*) with *p* ∈ (0, 1] are typical *p*-quasi-Banach spaces. Also, when *φ* is of uniformly lower type *p* ∈ (0, 1], the space *H*_*A*_^*φ*^(ℝ^*n*^) is a *p*-quasi-Banach space. Moreover, according to the Aoki-Rolewicz theorem (see [47] or [48]), any quasi-Banach space is, in essential, a *γ*-quasi-Banach space, where *γ*≔{log⁡_2_(2*K*)}^−1^.

For any given *γ*-quasi-Banach space *ℬ*_*γ*_ with *γ* ∈ (0, 1] and a sublinear space *𝒴*, an operator *T* from *𝒴* to *ℬ*_*γ*_ is said to be *ℬ*_*γ*_ *-sublinear* if, for any *f*, *g* ∈ *𝒴* and complex numbers *λ*, *ν*, it holds true that

(153) $$\begin{array}{c} {||T(\lambda f+\nu g)||}_{{ℬ}_{\gamma }}^{\gamma }\leq {\left|\lambda \right|}^{\gamma }{||T(f)||}_{{ℬ}_{\gamma }}^{\gamma }+{\left|\nu \right|}^{\gamma }{||T(g)||}_{{ℬ}_{\gamma }}^{\gamma } \end{array}$$

and ||*T*(*f*)−*T*(*g*)||_*ℬ*_*γ*__ ≤ ||*T*(*f*−*g*)||_*ℬ*_*γ*__.

We remark that, if *T* is linear, then *T* is *ℬ*_*γ*_-sublinear. Moreover, if *ℬ*_*γ*_≔*L*_*w*_^*q*^(ℝ^*n*^) with *q* ∈ [1, *∞*], *w* is a Muckenhoupt *A*_*∞*_ weight, and *T* is sublinear in the classical sense, then *T* is also *ℬ*_*γ*_-sublinear.

**Theorem 44**

Let (*φ*, *q*, *s*) be an admissible triplet. Assume that *φ* is an anisotropic growth function satisfying the uniformly locally dominated convergence condition and being of uniformly upper type *γ* ∈ (0, 1] and *ℬ*_*γ*_ a quasi-Banach space. If one of the following holds true,

(i) *q* ∈ (*q*(*φ*), *∞*), and *T* : *H*_*A*,*fin*_^*φ*,*q*,*s*^(ℝ^*n*^) → *ℬ*_*γ*_ is a *ℬ*_*γ*_-sublinear operator such that
  (154) $$\begin{array}{c} S≔\sup\left\{{||T(a)||}_{{ℬ}_{\gamma }}:a\;is\;any\;\left(\varphi ,q,s\right)\text{-}atom\right\}<\infty , \end{array}$$
(ii) *T* is a *ℬ*_*γ*_-sublinear operator defined on continuous (*φ*, *∞*, *s*)-atoms such that
  (155) S≔sup
⁡{||T(a)||ℬγ:a  is  any  continuous (φ,∞,s)-atom}<∞,
 then *T* has a unique bounded *ℬ*_*γ*_-sublinear operator extension $\tilde{T}$ from *H*_*A*_^*φ*^(ℝ^*n*^) to *ℬ*_*γ*_.

**Proof**

Suppose that the assumption (i) holds true. For any *f* ∈ *H*_*A*,fin_^*φ*,*q*,*s*^(ℝ^*n*^), by Theorem 42(i), there exist complex numbers {*λ*_*j*_}_*j*=1_^*k*^ and (*φ*, *q*, *s*)-atoms {*a*_*j*_}_*j*=1_^*k*^ supported in balls {*x*_*j*_ + *B*_*ℓ*_*j*__}_*j*=1_^*k*^ such that *f* = ∑_*j*=1_^*k*^ *λ*_*j*_ *a*_*j*_ pointwise. By Remark 31(i), we know that

(156) Λ~q({λj}j=1k) =inf⁡{λ∈(0,∞):     ∑j=1kφ(xj+Bℓj,|λj|||χxj+Bℓj||Lφ(ℝn)−1λ)≤1} ≲||f||HAφ(ℝn).

Since *φ* is of uniformly upper type *γ*, it follows that there exists a positive constant *C*_*γ*_ such that, for all *x* ∈ ℝ^*n*^, *s* ∈ [1, *∞*), and *t* ∈ (0, *∞*),

(157) $$\begin{array}{c} \varphi \left(x,st\right)\leq C_{\gamma }s^{\gamma }\varphi \left(x,t\right). \end{array}$$

If there exists *j*_0_ ∈ {1,…, *k*} such that *C*_*γ*_|*λ*_*j*_0__|^*γ*^ ≥ ∑_*j*=1_^*k*^|*λ*_*j*_|^*γ*^, then

(158) ∑j=1kφ(xj+Bℓj,|λj|||χxj+Bℓj||Lφ(ℝn)−1Cγ−1/γ(∑j=1k|λj|γ)1/γ)  ≥φ(xj0+Bℓj0,||χxj0+Bℓj0||Lφ(ℝn)−1)=1.

Otherwise, it follows, from (157), that

(159) ∑j=1kφ(xj+Bℓj,|λj|||χxj+Bℓj||Lφ(ℝn)−1Cγ−1/γ(∑j=1k|λj|γ)1/γ) ≳∑j=1k|λj|γ∑j=1k|λj|γφ(xj+Bℓj,||χxj+Bℓj||Lφ(ℝn)−1)∼1,

which implies that

(160) $$\begin{array}{c} {\left(\overset{k}{\underset{j=1}{\sum }}{\left|\lambda_{j}\right|}^{\gamma }\right)}^{1/\gamma }\leq C_{\gamma }^{1/\gamma }{\tilde{\Lambda }}_{q}\left({\left\{\lambda_{j}\right\}}_{j=1}^{k}\right)≲{||f||}_{H_{A}^{\varphi }({\mathbb{R}}^{n})}. \end{array}$$

Therefore, by the assumption (i), we obtain

(161) ||Tf||ℬγ=||T(∑j=1kλjaj)1/γ||ℬγ≲(∑j=1k|λj|γ)1/γ≲||f||HAφ(ℝn).

Since *H*_*A*,fin_^*φ*,*q*,*s*^(ℝ^*n*^) is dense in *H*_*A*_^*φ*^(ℝ^*n*^), a density argument then gives the desired conclusion.

Suppose now that the assumption (ii) holds true. Similar to the proof of (i), by Theorem 42(ii), we also conclude that, for all *f* ∈ *H*_*A*,fin_^*φ*,*∞*,*s*^(ℝ^*n*^)∩*ℂ*(ℝ^*n*^), ||*T*(*f*)||_*ℬ*_*γ*__≲||*f*||_*H*_*A*_^*φ*^(ℝ^*n*^)_. To extend *T* to the whole *H*_*A*_^*φ*^(ℝ^*n*^), we only need to prove that *H*_*A*,fin_^*φ*,*∞*,*s*^(ℝ^*n*^)∩*ℂ*(ℝ^*n*^) is dense in *H*_*A*_^*φ*^(ℝ^*n*^). Since *H*_*φ*,fin_^*p*,*∞*,*s*^(ℝ^*n*^; *A*) is dense in *H*_*A*_^*φ*^(ℝ^*n*^), it suffices to prove *H*_*A*,fin_^*φ*,*∞*,*s*^(ℝ^*n*^)∩*ℂ*(ℝ^*n*^) is dense in *H*_*φ*,fin_^*p*,*∞*,*s*^(ℝ^*n*^; *A*) in the quasinorm ||·||_*H*_*A*_^*φ*^(ℝ^*n*^)_. Actually, we only need to show that *H*_*A*,fin_^*φ*,*∞*,*s*^(ℝ^*n*^)∩*ℂ* ^*∞*^(ℝ^*n*^) is dense in *H*_*A*,fin_^*φ*,*∞*,*s*^(ℝ^*n*^) due to Theorem 42.

To see this, let *f* ∈ *H*_*A*,fin_^*φ*,*∞*,*s*^(ℝ^*n*^). Since *f* is a finite linear combination of functions with bounded supports, it follows that there exists *l* ∈ *ℤ* such that supp⁡*f* ⊂ *B*_*l*_. Take *ψ* ∈ *𝒮*(ℝ^*n*^) such that supp⁡*ψ* ⊂ *B*_0_ and ∫_ℝ^*n*^_ *ψ*(*x*)*dx* = 1. By (7), it is easy to show that supp⁡(*ψ*_*k*_∗*f*) ⊂ *B*_*l*+*σ*_ for any −*k* < *l*, and *f*∗*ψ*_*k*_ has vanishing moments up to order *s*, where *ψ*_*k*_(*x*)≔*b* ^*k*^ *ψ*(*A* ^*k*^ *x*) for all *x* ∈ ℝ^*n*^. Hence, *f*∗*ψ*_*k*_ ∈ *H*_*A*,fin_^*φ*,*∞*,*s*^(ℝ^*n*^)∩*ℂ* ^*∞*^(ℝ^*n*^).

Likewise, supp⁡(*f* − *f*∗*ψ*_*k*_) ⊂ *B*_*l*+*σ*_ for any −*k* < *l*, and *f* − *f*∗*ψ*_*k*_ has vanishing moments up to order *s*. Take any *q* ∈ (*q*(*φ*), *∞*). By [6, Proposition 2.9  (ii)] and the fact that *φ* satisfies the uniformly locally dominated convergence condition, we know that

(162) ||f−f∗ψk||Lφq(Bl+σ)⟶0 as  k⟶∞

and hence *f* − *f*∗*ψ*_*k*_ = *c*_*k*_ *a*_*k*_ for some (*φ*, *q*, *s*)-atom *a*_*k*_, where *c*_*k*_ is a constant depending on *k* and *c*_*k*_ → 0 as *k* → *∞*. Thus, we obtain ||*f*−*f*∗*ψ*_*k*_||_*H*_*A*_^*φ*^(ℝ^*n*^)_ → 0 as *k* → *∞*. This finishes the proof of Theorem 44.
